# Test–retest performance of [ ^18^ F]MK-6240 tau burden and relative delivery indices in cognitively normal older subjects using PET/MRI

**DOI:** 10.1162/imag_a_00402

**Published:** 2024-12-20

**Authors:** Cristina Lois, Jessie Fanglu Fu, Andrew N. Salvatore, Arun H. Garimella, Derek Huell, Hasan Sari, David Izquierdo Garcia, Nathaniel D. Mercaldo, Bradford Dickerson, Keith A. Johnson, Ciprian Catana, Julie C. Price

**Affiliations:** Department of Radiology, Massachusetts General Hospital, Boston, MA, United States; Gordon Center for Medical Imaging, Division of Nuclear Medicine and Molecular Imaging, Boston, MA, United States; Harvard Medical School, Boston, MA, United States; Athinoula A. Martinos Center for Biomedical Imaging, Charlestown, MA, United States; Department of Neurology, Massachusetts General Hospital, Boston, MA, United States

**Keywords:** [ ^18^ F]MK-6240 test–retest, Alzheimer’s disease, tau imaging, positron emission tomography, quantification, extracerebral signal.

## Abstract

Accurate interpretation of quantitative positron emission tomography (PET)outcomes hinges on understanding the test–retest variability (T-RT).Previous studies of the tau-PET ligand [^18^F]MK-6240 reported adequateT-RT performance of tau burden estimates over a short-term 21-day and over alonger-term 6-month T-RT period, primarily involving Alzheimer’s disease(AD) and cognitively normal (CN) subjects, respectively. However, several T-RTcharacteristics have not yet been reported, particularly in older CN (oCN)subjects. Here, we investigate the short-term T-RT performance of dynamic[^18^F]MK-6240 outcomes in a group largely consisting of oCN. Wereport T-RT for uptake in potential reference regions, for extracerebraloff-target signal, and for estimates of tau burden and relative delivery indicesin tau-bearing target regions. Eight participants (7 oCN, 1 AD) underwentbaseline dynamic [^18^F]MK-6240 PET/MRI (Biograph mMR) and a retestfollow-up PET/MRI scan within approximately 3 weeks. T-RT was evaluated usingabsolute percentage differences and intraclass correlation coefficients (ICC) inthree groups of regions: (1) potential reference regions usingstandardized-uptake values 90–110 minutes post-injection(SUV_90–110_); (2) target regions using SUV ratios(SUVR_90–110_), distribution volume ratios (DVR), andrelative delivery (R_1_); and (3) extracerebral region usingSUVR_90–110_. A voxel-based partial volume correction (PVC)was applied. T-RT was evaluated with and without PVC. In oCN subjects, theSUV_90–110_T-RT in the evaluated reference regions rangedfrom 6 to 11% (ICC > 0.9); target region T-RT was similar forSUVR_90–110_(4–9%, ICC: 0.62–0.97), DVR(3–10%, ICC: 0.66–0.92), and R_1_(3–14%, ICC:0.52–0.97). PVC had minimal impact on reference regionSUV_90–110_T-RT, but increased target region T-RTvariability (SUVR_90–110_: 10–26%; DVR: 6–22%;R_1_: 4–20%). Extracerebral SUVR_90–110_exhibited higher T-RT variability (~12%, ICC: 0.85) than other target regions(average 6%) and increased to ~15% after PVC. Our findings are consistent withprevious reports and provide further evidence of acceptable[^18^F]MK-6240 T-RT in low-signal oCN subjects. Our results suggest[^18^F]MK-6240 is suitable for detecting early tau deposition andlongitudinal changes over time, and further support the viability of[^18^F]MK-6240 R_1_to evaluate longitudinal changes inperfusion. PVC increased T-RT variability in tau burden and R_1_outcomes. Notably, the extracerebral signal exhibited higher T-RT variabilitythan other target and reference regions and may affect their signal.

## 1 Background

Positron emission tomography (PET) imaging has greatly advanced our understanding ofthe emergence, distribution, and longitudinal accumulation of amyloid-beta(Aβ) plaques and tau-containing neurofibrillary tangles (NFT) in the brainsof living individuals, in the context of both ageing and Alzheimer’s disease(AD). However, the PET detection of early deposition of hyperphosphorylatedpaired-helical filament tau in entorhinal and mesial temporal cortical areas poses adistinct set of challenges compared with those associated with the detection ofearly deposition of fibrillar Aβ plaques in the cortex (Villemagne et al., 2012). A well-known challenge oftau-PET imaging is the presence of off-target signal, which has been found toincrease with age (Baker et al., 2019;Tissot et al., 2022) and exhibit sex-relateddifferences (Scott et al., 2023;Smith et al., 2021). The off-target signal isprobably best understood for [^18^F]flortaucipir and[^18^F]THK-5351. Neuropathological and PET kinetic studies havedemonstrated off-target signal of [^18^F]flortaucipir in the basal gangliaand choroid plexus (Baker et al., 2019;Flores et al., 2022;López-González et al., 2022;Marquié et al., 2017;Tissot et al., 2022) and have revealed that[^18^F]THK-5351 has significant binding to the MAO-B enzyme complex(Ng et al., 2017). A second generation oftau-PET radioligands was developed in the interest of mitigating off-target signaland improving PET signal-to-noise ratios. These radioligands aimed to improve thedetection of low tau levels and gain a deeper understanding of its emergence,distribution, and longitudinal accumulation. Comprehensive information about thecharacteristics of these radioligands has been summarized in various review articles(Lee et al., 2023;Lois et al., 2019;Saint-Aubert et al., 2017) and comparative clinical evaluations (Bischof et al., 2021;Yap et al., 2021).

[^18^F]MK-6240 is one of the second-generation tau-selective radioligandswith improved in vivo imaging characteristics (Betthauser, Cody, et al., 2019;Guehl etal., 2019;Hostetler et al., 2016;Koole et al., 2020) that is gaining wideuse in clinical studies. Numerous [^18^F]MK-6240 PET studies have reportedstrong signal-to-noise ratio in tau-bearing areas, showing utility for the detectionof early and late accumulation of tau, as well as for disease staging in individualsranging from preclinical to symptomatic AD (Bennacefet al., 2017;Betthauser, Koscik, et al.,2019;Pascoal et al., 2020a,2020b). However, a substantial off-targetbinding signal is evident in the extracerebral space (Fu et al., 2023;Mertenset al., 2021;Vanderlinden et al.,2022) in about 50% of subjects (Fu etal., 2023). Neuropathological evaluations of human tissue have verified[^18^F]MK-6240 binding to melanin-containing cells, such as those foundin the meninges within the extracerebral space (Aguero et al., 2019). The extracerebral signal has the potential tospill into the brain, including cortical and reference region areas, and thusconfound quantification across cognitively normal (CN) to advanced AD subjects. Tominimize this effect, several studies have selected various reference regions tonormalize the PET measurement outcomes. Despite no consensus about which is optimal,some commonly used reference regions include grey matter-based regions such as thecerebellar grey matter (with or without erosion of the outer regional voxels) or theinferior cerebellar grey matter; white matter-based regions such as the cerebralwhite matter (with or without erosion of the outer regional voxels); and mixedregions such as the pons or whole cerebellum (Fu etal., 2023;Gogola et al., 2020;Guehl et al., 2019;Lohith et al., 2019;Pascoal et al., 2018,2020a,^2021^;Salinas et al., 2019;Shuping et al.,2023). Other investigators have proposed less common reference regions,such as the cerebellar white matter (Becker et al.,2023). Due to the spill-in contamination from the extracerebral signal toreference and target regions, the extracerebral signal has, consequently, thepotential to impact the short- and long-term reproducibility of PET outcomemeasures.

Recently, the suitability of [^18^F]MK-6240 dynamic imaging to providesurrogate measurements of cerebral perfusion has been investigated. Deficits incerebral perfusion have been reported in older subjects (Leenders et al., 1990;H.Lu et al., 2011) and in subjects with AD (Austin et al., 2011;Johnson &Albert, 2000) or other tauopathies (Hu etal., 2010), and are important for understanding the pathogenesis of thesediseases and ageing. Although cerebral perfusion can be measured using MRI-basedmethods such as arterial spin labelling (ASL) or with the gold standard[^15^O]water PET, these require a PET/MRI scanner or a separatescanning session, which leads to added subject burden and increased logisticaldifficulties. This has motivated investigations of whether the early phase ofamyloid or tau PET imaging can provide surrogate measurements of cerebral perfusion(Bilgel et al., 2019;Chen et al., 2015;Ottoyet al., 2019;Rodriguez-Vieitez et al.,2016;Visser et al., 2020). Tworecent studies support the use of the early phase of [^18^F]MK-6240 dynamicdata to derive robust estimates of relative delivery (R_1_) as aquantitative index of relative cerebral perfusion (Fu et al., 2022;Guehl et al.,2023), potentially allowing dual-imaging assessments of tau and cerebralperfusion. However, [^18^F]MK-6240 R_1_variability has not yetbeen reported.

Test–retest (T-RT) variability informs about how different the outcomemeasurements are when data are obtained repeatedly on the same subject and undersimilar conditions (i.e., within-subject variability). Understanding the T-RTvariability is crucial for the accurate interpretation of quantitative results. Thisis particularly relevant for longitudinal studies that involve multiple scans overtime, such as the investigation of the natural time course of tau accumulation, orthe effects of therapy in drug intervention studies where small changes in PETsignal need to be detected. Given its relevance, T-RT is one of the properties oftenevaluated for novel PET tracers (Bullich et al.,2020;Devous et al., 2018;Finnema et al., 2017;Salinas et al., 2019;Timmers et al., 2019;Vanderlinden etal., 2022).

Two recent studies reported [^18^F]MK-6240 T-RT results for subjects withdifferent characteristics. First, in an AD subject-dominant sample consisting of 12AD (65 ± 1 years of age) and 3 CN (55 ± 7 years of age) subjects,Salinas et al. (2019)determined T-RTfrom dynamic [^18^F]MK-6240 PET imaging performed within 21 days. Acrossall subjects, the average T-RT percentage differences in tau-bearing regions wereapproximately 21%, 14%, and 6% for the total distribution volume (V_T_),binding potential (BP_ND_), and late-frame standardized-uptake-value ratio(SUVR_90–120_) PET outcomes, respectively. However, the T-RTvariability of the extracerebral signal was not reported. Second, in a sampleconsisting of 10 CN subjects (56 ± 12 years of age),Vanderlinden et al. (2022)reported a long-term 6-month[^18^F]MK-6240 late-frame SUVR_90–120_T-RT of 2.4± 2.8% in whole-brain grey matter. The authors also reported that, at grouplevel, the extracerebral uptake was not significantly different at the 6-monthfollow-up compared with baseline (extracerebral SUVR_90–120_T-RT:4.4 ± 20%), nor at a 2-year follow-up for a group of 10 amnestic mildcognitive impairment subjects (extracerebral SUVR_90–120_T-RT: 7.9± 19%). The extracerebral signal was found to correlate with age(*r*’s = −0.48;*p*<0.0001) and to be higher in women.

Although the two previous studies included CN subjects, the average age of thoseparticipants was 56 years. Estimating the T-RT variability in older CN subjects(>65 years of age) who may be at higher risk of AD may, however, be criticalfor detecting the emergence of early tau deposition. In addition, these studies didnot evaluate the T-RT characteristics of the reference regions, an important step tounderstanding the variability in normalized target region outcomes. Furthermore,although recent studies have shown that the relative radioligand delivery indexR_1_estimated from dynamic [^18^F]MK-6240 acquisition mayprovide reliable estimates of relative perfusion (Fuet al., 2022;Guehl et al., 2023),the evaluation of the T-RT characteristics of [^18^F]MK-6240 R_1_is still needed in order to provide further support for dual-biomarker imaging toquantify both tau deposition and relative cerebral perfusion across the AD spectrum.Finally, to reduce the spill-in contamination from the extracerebral signal to thetarget and reference regions, outer voxel erosion and partial volume correction(PVC) techniques have been used (Fu et al.,2023;Mertens et al., 2021). It isknown that the processing methods and applied corrections may affect thequantification of the PET data and, thus, may impact the T-RT variability of PEToutcomes. However, the impact of PVC and reference region erosion on the T-RTcharacteristics of the [^18^F]MK-6240 uptake in the target and referenceregions is still unclear.

Here, we further evaluate the short-term T-RT characteristics of dynamic[^18^F]MK-6240 PET on a sample largely consisting of older CNindividuals (oCN, median age 66 years, interquartile range (IQR) [65,71]) studiedtwice within approximately 3 weeks. First, we evaluate the T-RT characteristics ineight potential reference regions commonly used in [^18^F]MK-6240 clinicalstudies (including grey matter-based, white matter-based, and mixed regions), and inthe extracerebral signal. Second, in 11 target regions (including areas of early andlate tau accumulation in AD), we assess the impact of using different referenceregions on the T-RT of late-frame SUVR. Third, we investigate the differences inregional T-RT values obtained from dynamic imaging outcomes (i.e., distributionvolume ratio, DVR) and from late-frame SUVR in target regions. Fourth, we extend thecurrent knowledge on [^18^F]MK-6240 dynamic imaging with the investigationof T-RT for R_1_. Lastly, we evaluate the impact of applying an iterativevoxel-based PVC on the T-RT of all analyzed outcome measurements.

## 2 Materials and Methods

### 2.1 Study participants

Eight participants (7 oCN, median age 66 years, IQR [65,71]; 1 AD, 54 years)underwent [^18^F]MK-6240 baseline PET/MR imaging (Test) and a follow-upPET/MR scan (Retest) within approximately 3 weeks (Test–Retest intervalIQR: 2 to 4 weeks). Nine additional subjects underwent baseline PET/MR only: 4young controls (yCN, median age 26 years, IQR [25,29]), 3 oCN, and 2 ADparticipants. These additional subjects were included in baseline analyses only,to facilitate comparisons with previously reported results and forcompleteness.

Subject characteristics are summarized inTable1. All participants in this study self-identified as non-HispanicWhite, except three oCN participants (one African American, one Asian, and onemixed White/Asian). APOE genotype and amyloid status were not available for mostsubjects. The study was approved by the local Institutional Review Board. The ADparticipants were recruited from the Massachusetts General Hospital (MGH)Neurology Units and Frontotemporal Disorders Unit after receiving a diagnosis ofprobable AD of mild severity. The AD diagnosis was performed by an experiencedneurologist at MGH specialized in dementia and was based on memory complaintsand functional impairment, as determined by a clinical interview with thesubject and an informant. The severity of these symptoms met the criteria forprobable AD of mild severity, as defined by the National Institute ofNeurological and Communicative Disorders and Stroke/Alzheimer’s Diseaseand Related Disorders Association (McKhann etal., 2011). CN participants were recruited from the local community.All subjects underwent neurological evaluations using the Mini-Mental StateExamination (MMSE). For the CN participants, assessment of normal cognition wasobtained through MMSE (MMSE > 25 for oCN and MMSE > 27 for yCN).Prior to enrollment, all CN subjects provided written informed consent toparticipate in the study, and all AD subjects assented to participate in thestudy with their study partner’s consent.

**Table 1.** Participant demographics and cognitive information.

	Test PET			Retest PET	
	yCN	oCN	AD	oCN	AD
n	4	10	3	7	1
T-RT interval (d)	—	—	—	23 [13,29]	30
Age (y)	26 [25,29]	69 [66,71]	55 [55,60]	66 [65,71]	54
Sex (F/M)	2 / 2	3 / 7	1 / 2	2 / 5	0 / 1
MMSE	29 [29,29]	30 [28,30]	18 [18,20]	29 [28,30]	22
Race
non-Hispanic White	4	7	3	6	1
African American	-	1	-	1	-
Asian	-	1	-	-	-
White/Asian	-	1	-	-	-

Subjects include young controls (yCN), older controls (oCN), andAlzheimer’s disease subjects (AD). Most participants in thisstudy self-identified as non-Hispanic White. APOE genotype andamyloid status were not available for most subjects. Continuousvariables are summarized as median [interquartile range].

### 2.2 Image acquisition and processing

#### 2.2.1 PET/MRI data acquisition

All subjects underwent dynamic Test PET following the intravenousadministration of an injection of 185 ± 15 MBq of[^18^F]MK-6240 (molar activity: 177 ± 70 GBq/μmol atthe end of the synthesis). Scans were performed on a whole-body Biograph-mMRscanner (Siemens Healthineers), which allows the simultaneous acquisition ofPET and MR images (Delso et al.,2011). PET data were acquired for 120 minutes divided into 2 segments(0–65 min and 80–120 min) with a 15-minute break during whichthe subjects were allowed to leave the scanner. The subset of subjects whounderwent Retest [^18^F]MK-6240 PET/MRI followed the same imagingprotocol (injected dose 186 ± 13 MBq). Simultaneous MR imaging wasperformed during each PET scan. A T1-weighted 3-dimensionalmagnetization-prepared rapid gradient-echo (MPRAGE) image was acquired atthe beginning of each segment of the PET scan (i.e., before and after thebreak) for the purpose of anatomical localization, exclusion of incidentalpathology, and generation of attenuation correction maps.

#### 2.2.2 MRI data processing

MPRAGE images were corrected for MR intensity inhomogeneity using N4ITK17(Tustison et al., 2010) and weredenoised using value thresholding and masking using Slicer3d (https://www.slicer.org) (Fedorov et al., 2012). For eachsubject, a single MPRAGE image was selected for automated volumetricsegmentation of cortical and subcortical brain structures, based on theleast evidence of subject motion on visual inspection (if visually similar,the baseline MPRAGE was selected). Using FreeSurfer (version 6,http://surfer.nmr.mgh.harvard.edu/), the MPRAGE image wassegmented into tissue types and used to classify anatomical brain volumes(Fischl et al., 2002,^2004^). Additional processing generatedthe extracerebral segmentations by dilating the pial surface by 5 mm outwardperpendicularly to the cortical surface, as described previously (Fu et al., 2023). The inferiorcerebellar grey matter region was obtained using the SUIT cerebellartemplate (Diedrichsen, 2006), reversenormalized to the subject’s MPRAGE space (Baker et al., 2017), and subtracted from theFreeSurfer-generated cerebellar grey matter.

The MPRAGE image was registered to the Test and Retest PET images, and thecorresponding transformation matrix was applied to the regionalsegmentations using FSL (FMRIB Software Library,https://fsl.fmrib.ox.ac.uk/fsl/) (Woolrich et al., 2009). The segmented regions of interest (ROIs)were used for regional sampling of the PET images and generation of regionaltime-activity curves (TACs). Identical ROIs were applied to the Test andRetest scans for each subject.

#### 2.2.3 PET data processing

Dynamic PET data were divided into frames (29 frames for the first segment: 6x 10 s, 6 x 20 s, 2 x 30 s, 2 x 60 s, 2 x 120 s, and 11 x 300 s; and 8frames for the second segment: 8 x 300 s) and were reconstructed using anordered-subset expectation maximization algorithm (3 iterations, 21 subsets,344 × 344 × 127 image matrix, with 2.1 mm in-plane pixel sizeand 2.0 mm slice thickness; 4 mm Gaussian filter). PET images were correctedfor dead time, decay, scatter, and attenuation. For each PET segment,attenuation maps were estimated from the respective MPRAGE image through acombination of intensity- and prior-based tissue segmentation and atlasregistration, as described previously (Izquierdo-Garcia et al., 2014). Motion correction was appliedthrough frame-by-frame realignment of the reconstructed data using FSL(Woolrich et al., 2009). Each PETsegment was individually motion corrected, and subsequently, the secondsegment was registered and concatenated to the first.

PVC was performed using the iterative Yang method (Erlandsson et al., 2012;Thomas et al., 2016;Y. Lu et al., 2021) (10 iterations), anadaptation of the Yang method (Yang et al.,1996) that applies a correction factor iteratively for eachvoxel. Briefly, a regional mean image is created by averaging the PETactivity values in each FreeSurfer segmentation for each PET frame. Apartial volume correction map is generated iteratively as the ratio of thenon-smoothed regional mean image and the regional mean image smoothed by a 5mm Gaussian kernel (value selected based on our scanner performance;Delso et al., 2011). The finalcorrection map is applied to the dynamic PET data at the voxel level.

Standardized uptake-value (SUV_90–110_, g/mL) images werecalculated for data acquired 90−110 min post-tracer injection byaveraging the corresponding four 5-min reconstructed frames and normalizingby body weight and injected dose.

#### 2.2.4 Quantitative and statistical analysis

As described above, ROIs were defined on the individual MPRAGE images and,subsequently, used to sample PET images and obtain regional TACs andSUV_90–110_values. For each subject, left and righthemisphere ROIs were averaged and analyzed together. Twenty a prioriselected regions were analyzed:

1. Reference regions. Eight regions (previously used for[^18^F]MK-6240 and other tau radioligands such as[^18^F]Flortaucipir) were examined: cerebellar greymatter (CerGM), cerebellar grey matter with a 3-mm erosion of theouter regional mask voxels (CerGM_3__mm_), inferior cerebellum (Inferior CerGM), cerebellarwhite matter (CerWM), cerebral white matter (WM), cerebral whitematter with 4-mm erosion (WM_4__mm_), whole cerebellum (WholeCer), and pons.
2. Target regions. Eleven regions known to have pathological tauaccumulation in various stages of AD were examined: entorhinalcortex, amygdala, hippocampus, fusiform, inferior temporal cortex,precuneus, insula, precentral gyrus, rostral middle frontal, lateraloccipital cortex, and a meta-temporal cortical composite (comprisingthe entorhinal, parahippocampus, amygdala, fusiform, inferior, andmiddle temporal cortex).
3. Extracerebral region.

The following PET outcome measures were evaluated: (1) For reference regions:SUV_90–110_; (2) For target regions:SUV_90–110_, SUV-ratios (SUVR_90–110_= SUV_TARGET_/SUV_REFERENCE_), distribution volumeratio (DVR) computed using Multilinear Reference-Tissue Model 2 (Ichise et al., 2003) (MRTM2; t*= 30 min, k2’ = 0.04;Betthauser, Cody, et al., 2019), and relative delivery(R_1_= K_1_TARGET_/K_1_REFERENCE_)estimated using Simplified Reference-Tissue Model (Lammertsma & Hume, 1996) (SRTM); (3) Forextracerebral region: SUV_90–110_andSUVR_90–110_. All outcome measures were computed forboth non-PVC and PVC data.

The variability of the outcome measures of interest was evaluated usingabsolute percentage T-RT differences, calculated as$T-RT\left(\%\right)=200*\frac{\left|Test_{outcome}-Retest_{outcome}\right|}{Test_{outcome}+Retest_{outcome}}$, and Bland–Altman plots in subjects with Retest PET(n = 8: 7 oCN, 1 AD). To further evaluate the within-subjectvariability, intraclass correlation coefficients (ICC, two-way model) werecalculated for each ROI in the T-RT oCN group only (n = 7, the ADsubject was excluded to avoid an increase in the between-subjectsvariability explained by disease). ICC values less than 0.5 were consideredpoor reliability, between 0.5 and 0.75 moderate, between 0.75 and 0.9 good,and greater than 0.90 were considered excellent reliability (Koo & Li, 2016).

Continuous demographic variables were reported as medians with interquartileranges (IQR) to address the small sample size. However, group-level PEToutcome measures were expressed as mean ± standard deviation (SD) tofacilitate comparisons with existing literature. Baseline reference regionoutcome measures were compared across regions using theKruskal–Wallis test. Differences between Test and Retest data in eachregion were evaluated using a Wilcoxon signed-rank test. Associationsbetween regional SUVR_90–110_and DVR values were assessedusing Spearman’s correlation. Significance level was assessed at*p*= 0.05. No multiple comparison correctionswere applied.

Statistical analyses were performed using Python version 3.9 (https://www.python.org/),including the SciPy (Virtanen et al.,2020), Seaborn (Waskom,2021), Matplotlib (Hunter,2007), Pandas (McKinney,2010), and Pingouin (Vallat,2018) packages. PET image analyses were performed using Miakat(Gunn et al., 2016).

## 3 Results

### 3.1 Brain distribution: visual inspection

The pattern of brain [^18^F]MK-6240 uptake distribution was consistentwith previous studies (Betthauser, Cody, et al.,2019;Fu et al., 2023;Guehl et al., 2019;Pascoal et al., 2018) and with the expectedlocalization of tau across the AD spectrum (Braak & Braak, 1999;Kreislet al., 2022;Pascoal et al.,2020a), and was similar in the Test and Retest scans (Supplementary Fig.1).

All subjects exhibited some degree of meningeal or extracerebral signal, withseven of them presenting visually high extracerebral signal. We observed highinter-subject variability in extracerebral uptake, both in location andmagnitude. In the meningeal space, CN participants showed varying signal levels,from high (e.g., Subjects 6 and 7;Supplementary Fig. 1) to relatively low (e.g., Subjects 2and 3;Supplementary Fig.1). The meningeal signal distribution was non-uniform. Allparticipants displayed elevated signal in the meningeal space superior to thecerebellum. In all AD participants, the superior portion of the cerebellumshowed elevated signal due to spill-in from the high tau binding nearby corticalregions. For the CN participants, the extracerebral signal was generally morepronounced inferior to the cerebellum compared with superior to the cerebellum.Extracerebral uptake was also observed in the sinuses, with high inter-subjectvariability. Two participants (Subjects 1 and 7;Supplementary Fig. 1)exhibited notable differences between the Test and Retest scans in thisregion.

**Fig. 1.**
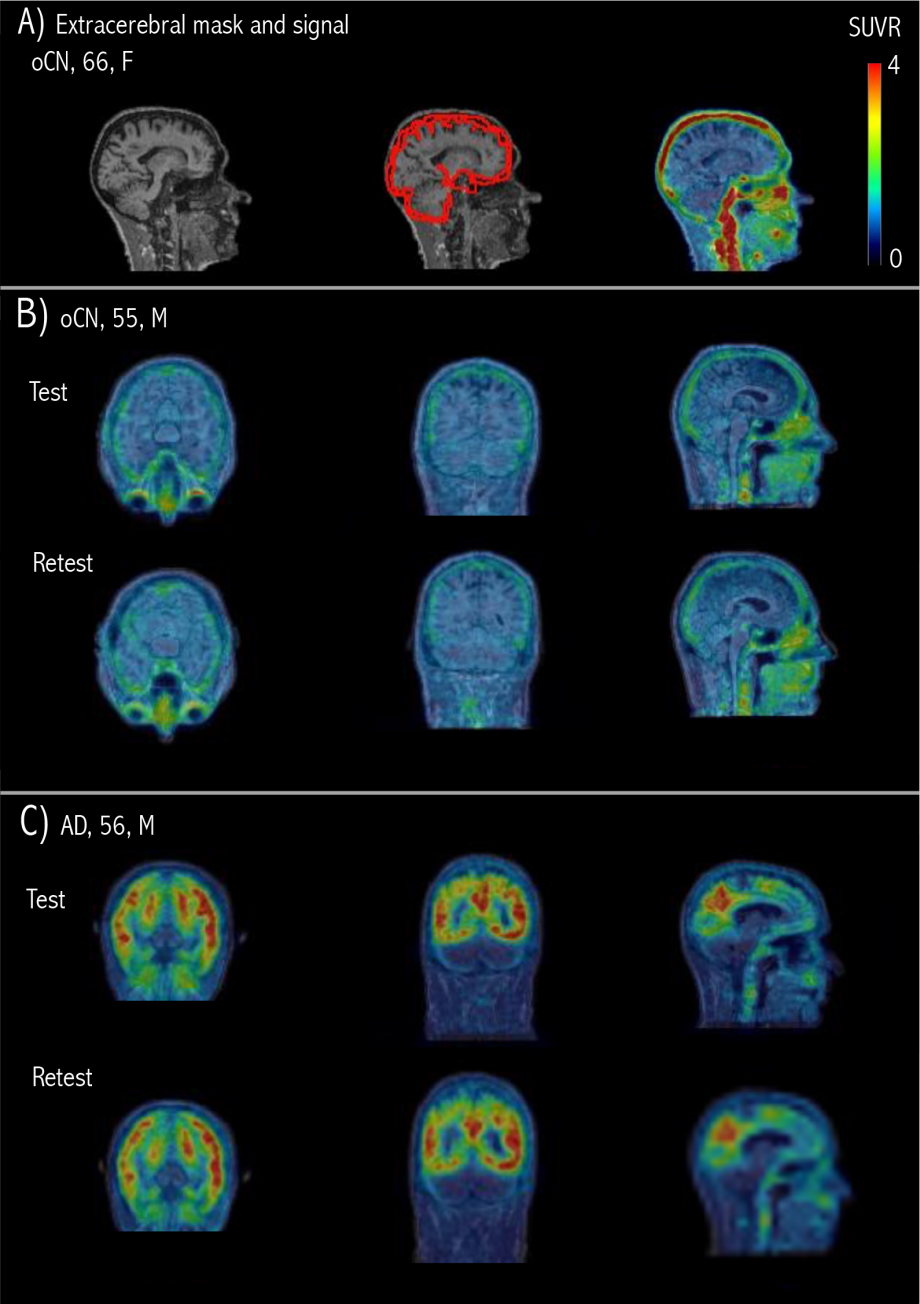
[^18^F]MK-6240 SUVR_90–110_PET images (usingCerGM as reference) overlaid on simultaneously acquired structural MRI.(A) Example oCN subject with high extracerebral signal and usedextracerebral mask (red). The mask was created by dilating theFreeSurfer-generated cortical segmentation by 2–5 mm outwardperpendicularly to the cortical surface. (B) Example oCN subject TestPET (top) and Retest PET (bottom), showing low cerebral uptake and amild but noticeable uptake in extracerebral regions, including themeninges. (C) Example AD subject Test PET (top) and Retest PET (bottom),showing high uptake in numerous cortical areas and noticeable uptake inextracerebral regions, including the meninges.

Figure 1Ashows an example oCN subject withhigh extracerebral signal in the PET SUVR_90–110_image (CerGMas the reference region) and the extracerebral segmentation. Illustrativeexamples of Test and Retest [^18^F]MK-6240 SUVR_90–110_PET images are shown for one oCN and one AD subject inFigure 1B and C, respectively. The oCN subject shows lowcerebral uptake and a mild but noticeable extracerebral signal. The AD subjectshows high uptake in numerous cortical areas and low extracerebral signal.

### 3.2 Reference regions: SUV _90–110_

#### 3.2.1 Reference region: SUV _90–110_ (no PVC)

**Fig. 2.**
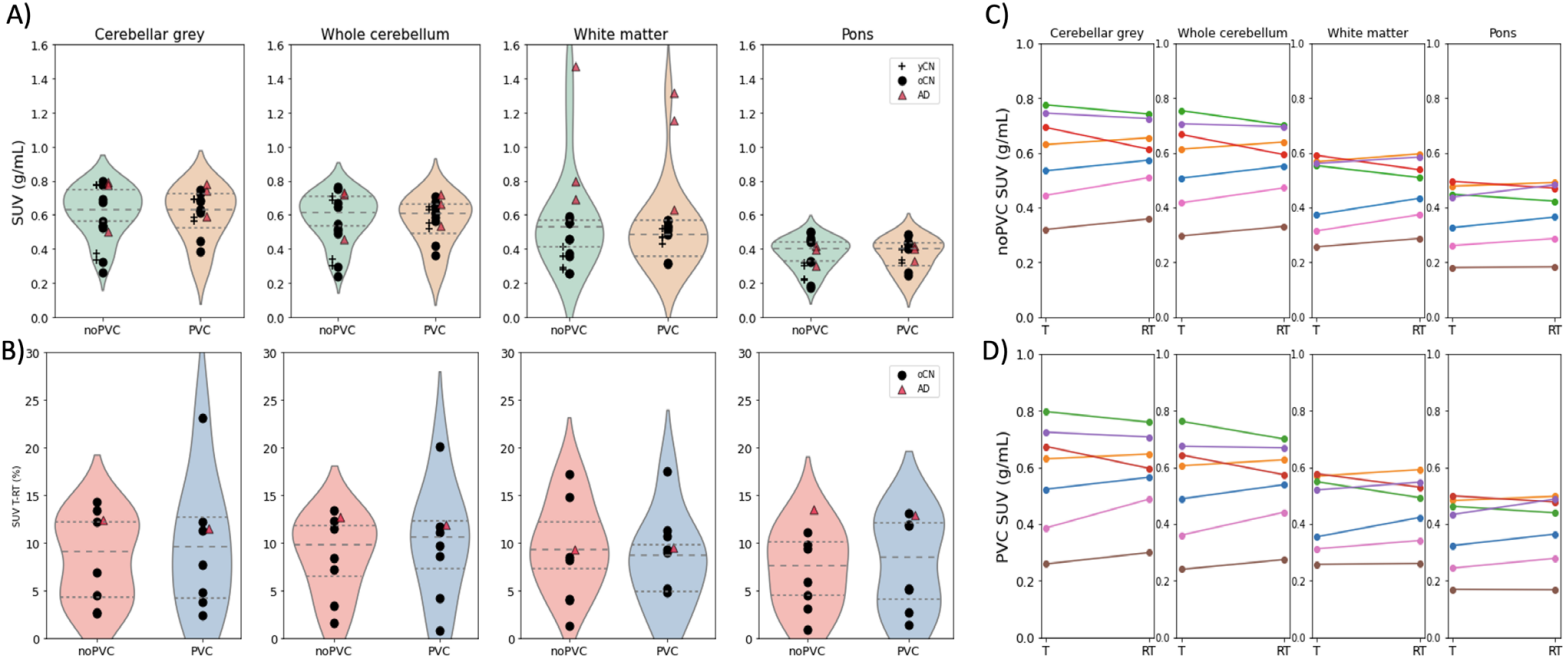
(A) Violin plots showing the baseline SUV_90–110_forthe selected reference regions, including cerebellar grey matter,whole cerebellum, cerebral white matter, and pons, with and withoutPVC (iterative Yang;Erlandsson etal., 2012). All subjects who underwent baseline PET areincluded (4 yCN, 10 oCN, and 3 AD). Mean regionalSUV_90–110_were similar across referenceregions and participant groups, except for the cerebral whitematter, which was roughly twofold greater in the AD group than inthe CN groups. The application of PVC resulted in a 17% decrease ofSUV_90–110_for the AD subjects in the cerebralwhite matter. (B) Violin plots showing the absolute percentage T-RTdifferences for the reference region SUV_90–110_with and without PVC. Only subjects undergoing both Test and RetestPET are included (7 oCN and 1 AD). (C) Individual Test (T) andRetest (RT) regional SUV_90–110_for the oCN group(n = 7) with no PVC, and (D) with PVC, demonstrating adequateT-RT characteristics in reference regionSUV_90–110_.

Table 2lists theSUV_90–110_values for the evaluated reference regions.The mean regional SUV_90–110_values ranged from 0.35 (pons)to 0.62 g/mL (inferior CerGM) in the CN groups, and from 0.38 (pons) to 1.11g/mL (WM) in the AD group. SUV_90–110_values were similaracross diagnostic groups for all evaluated reference regions except for thecerebral WM and WM_4__mm_, which were twofold higher in the AD group than in the CNgroups. The pons showed the lowest SUV_90–110_among allevaluated reference regions in both the CN and AD groups, although therewere no significant differences between any of these reference regions.Figure 2Ashows violin plotsvisualizing baseline SUV_90–110_for illustrative referenceregions, including the CerGM, WholeCer, WM, and pons.

**Table 2.** Baseline SUV_90–110_(no PVC) and correspondingSUV_90–110_T-RT (%).

Region	SUV (g/mL yCN, n = 4)	SUV (g/mL oCN, n = 10)	SUV T-RT (%, oCN, n = 7)	SUV (g/mL AD, n = 3)	SUV T-RT (%, AD, n = 1)
CerGM	0.60 ± 0.17	0.60 ± 0.14	7.8 ± 4.2	0.72 ± 0.11	12.5
CerGM _3 mm_	0.53 ± 0.18	0.55 ± 0.16	9.5 ± 4.0	0.59 ± 0.15	9.3
Inferior CerGM	0.62 ± 0.18	0.61 ± 0.14	9.3 ± 5.4	0.68 ± 0.10	8.1
WholeCer	0.57 ± 0.16	0.56 ± 0.14	8.1 ± 3.9	0.66 ± 0.11	12.7
CerWM	0.39 ± 0.11	0.46 ± 0.14	10.9 ± 6.8	0.47 ± 0.12	12.8
WM	0.42 ± 0.10	0.47 ± 0.12	9.9 ± 4.7	1.11 ± 0.39	9.3
WM _4 mm_	0.36 ± 0.09	0.44 ± 0.11	9.5 ± 5.7	0.78 ± 0.29	9.8
Pons	0.35 ± 0.07	0.39 ± 0.10	6.5 ± 3.6	0.38 ± 0.05	13.5
Entorhinal	0.57 ± 0.07	0.66 ± 0.20	8.9 ± 5.5	1.26 ± 0.35	6.9
Amygdala	0.34 ± 0.07	0.46 ± 0.13	6.8 ± 3.4	0.94 ± 0.33	10.0
Hippocampus	0.37 ± 0.07	0.50 ± 0.16	7.3 ± 4.3	0.74 ± 0.12	3.6
Fusiform	0.57 ± 0.11	0.61 ± 0.15	9.6 ± 10.0	1.58 ± 0.59	6.5
Inf. Temporal	0.64 ± 0.11	0.65 ± 0.15	9.4 ± 9.3	1.77 ± 0.65	9.3
Rostral Mid. Frontal	0.58 ± 0.14	0.53 ± 0.11	11.4 ± 9.9	1.51 ± 0.81	5.7
Lateral Occipital	0.71 ± 0.16	0.68 ± 0.13	14.6 ± 8.2	1.37 ± 0.47	20.0
Precuneus	0.48 ± 0.11	0.52 ± 0.12	7.3 ± 4.8	1.89 ± 0.71	9.6
Insula	0.42 ± 0.10	0.47 ± 0.11	6.8 ± 5.8	0.98 ± 0.44	11.4
Precentral	0.53 ± 0.14	0.49 ± 0.11	10.7 ± 8.3	1.07 ± 0.36	8.8
Meta-temporal	0.60 ± 0.11	0.62 ± 0.14	9.2 ± 7.7	1.59 ± 0.56	10.4
Extracerebral	0.80 ± 0.11	0.77 ± 0.11	10.9 ± 10.1	0.91 ± 0.26	16.9

Only one AD subject underwent Retest PET. None of the yCNunderwent Retest PET and, therefore, T-RT data are unavailablefor this group. Values are expressed as mean±standarddeviation. The meta-temporal region is a composite ofentorhinal, parahippocampus, amygdala, fusiform, inferior, andmiddle temporal gyri.

#### 3.2.2 Reference region SUV _90–110_ (no PVC): T-RT and ICC

For the evaluated reference regions, the mean regionalSUV_90–110_T-RT variability ranged from 6 to 11% in theoCN group, and from 8 to 13% in the AD group (Table 2).Figure2Bshows violin plots representing SUV_90–110_T-RT for some illustrative reference regions. The ICC values were excellentfor all reference regions (≥0.89;Supplementary Fig.2). In oCN, among the evaluated reference regions, the ponsshowed the lowest SUV_90–110_T-RT variability (6.5%) andhighest ICC (0.97), while the CerWM showed the highestSUV_90–110_T-RT variability (10.9%) and lowest ICC(0.89). The Wilcoxon rank-sum test revealed no statistical differencesbetween the Test and Retest SUV_90–110_for any of theevaluated regions.Figure 2Cshowsindividual Test and Retest SUV_90–110_values in thoseselected regions.

#### 3.2.3 Reference region: Impact of PVC on SUV _90–110_ , SUV _90–110_ T-RT, and ICC

The iterative Yang PVC SUV_90–110_and corresponding T-RTvalues are provided inSupplementary Table 1. PVC SUV_90–110_valuesranged from 0.31 (pons) to 0.59 g/mL (CerGM and inferior CerGM) in the CNgroups, and from 0.37 (pons) to 0.92 g/mL (WM) in the AD group. Theapplication of PVC had a minor impact on the range ofSUV_90–110_for all evaluated reference regions, exceptfor the cerebral WM in the AD group, where PVC SUV_90–110_decreased by 17% compared with the non-PVC SUV_90–110_. Thecorresponding SUV_90–110_T-RT and ICC values were similarbetween PVC and non-PVC SUV_90–110_. The impact of applyingPVC to SUV_90–110_in the selected reference regions and onthe corresponding SUV_90–110_T-RT is illustrated inFigure 2A and B, respectively, and theTest and Retest PVC SUV_90–110_values for each oCN subjectare shown inFigure 2D.

### 3.3 Target regions: SUVR _90–110_ , DVR and R _1_

#### 3.3.1 Target regions: Impact of reference region selection on SUVR _90–110_ and SUVR _90–110_ T-RT

The impact of normalizing by different reference regions onSUVR_90–110_and SUVR_90–110_T-RT issummarized inSupplementary Table 2. In the CN groups, normalization by CerGMand Inferior CerGM resulted in the lowest SUVR_90–110_values, ranging from 0.58 (amygdala) to 1.14 (lateral occipital), while ponsresulted in the highest, ranging from 1.08 (amygdala) to 2.26 (lateraloccipital). In the AD group, normalization by WM and WM_4__mm_resulted in lower SUVR_90–110_values thanother reference regions, due to spill-in signal from grey matter to thewhite matter-based reference regions. Excluding WM and WM_4__mm_, in the AD group, CerGM and pons resulted in the lowest,respectively, highest SUVR_90–110_values, consistent withthe CN groups. In the CN groups, target region SUVR_90–110_T-RT variability was similar when using for normalization CerGM-basedreference regions (CerGM: 4–9%, CerGM_3__mm_: 5–10%, Inferior CerGM: 4–9%,), WholeCer(4–9%) or WM (3–11%), and slightly higher in WM_4__mm_(2–14%), pons (3–14%), and CerWM (6–15%).In addition, regardless of the reference region used, the rank orders of thetarget regions remained consistent in the AD subjects.

For the remaining analyses, we selected CerGM as the reference region, as itis one of the most used reference regions in the current literature (Bourgeat et al., 2023;Krishnadas et al., 2023;Vanderlinden et al., 2022), and all evaluatedreference regions resulted in similar T-RT SUVR_90–110_variability.

#### 3.3.2 Target regions: SUVR _90–110_ , DVR, and R _1_ (no PVC)

**Fig. 3.**
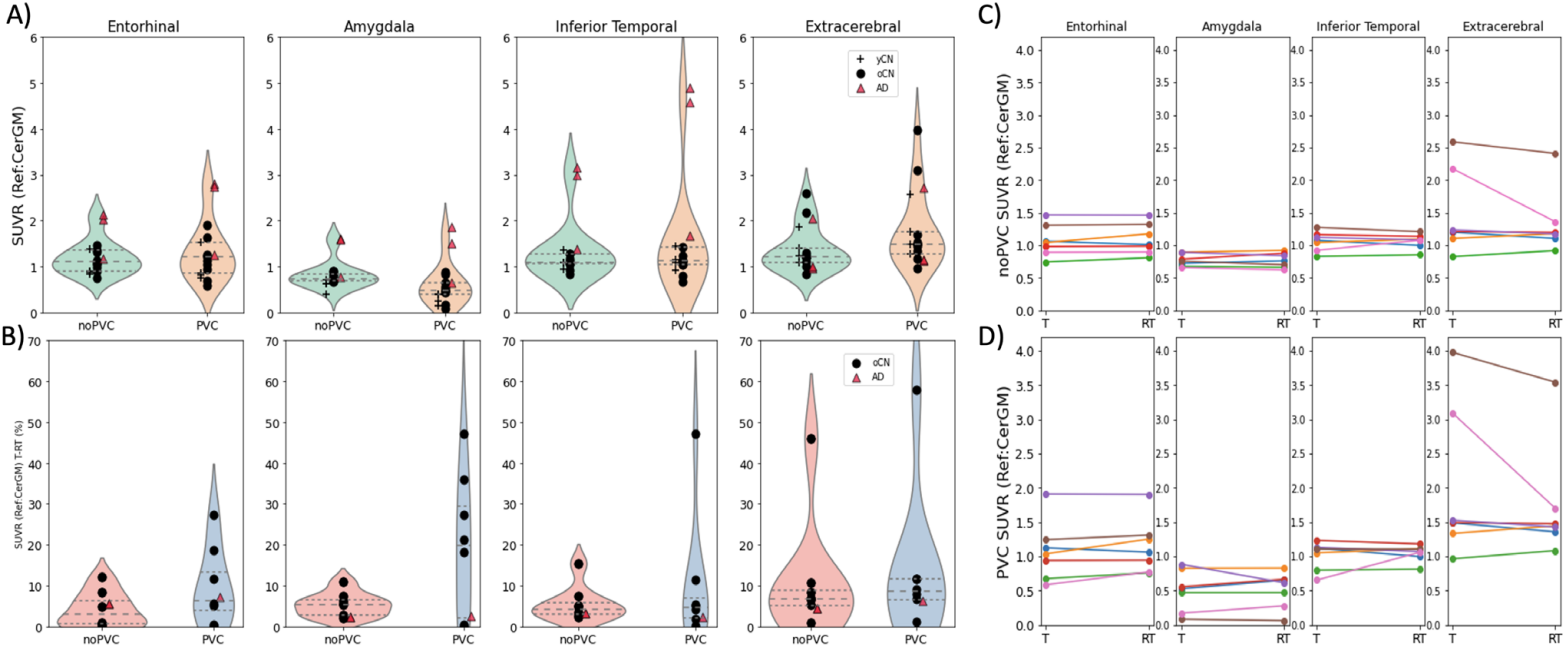
(A) Violin plots showing the baseline SUVR_90–110_for selected target regions (entorhinal, amygdala, and inferiortemporal cortex), and extracerebral signal, with and withoutiterative Yang PVC (Erlandsson etal., 2012). All subjects (4 yCN, 10 oCN, and 3 AD) areincluded. Mean regional SUVR_90–110_values weresimilar across regions for the CN groups but, as expected, werehigher in the AD group. Applying PVC increased the variance ofSUVR_90–110_in the CN and AD groups. (B)Corresponding SUVR_90–110_T-RT (%) for the sametarget regions. Only subjects undergoing both Test and Retest PETare included (7 oCN and 1 AD). The application of PVC resulted in anapproximately twofold increase of SUVR_90–110_T-RTvariability in the oCN group. (C) Individual Test and Retestregional SUVR_90–110_for the oCN group (n =7) with no PVC, and (D) with PVC in the target and extracerebralregions.

Table 3summarizes theSUVR_90–110_, DVR, and R_1_values obtainedusing CerGM as the reference region for the examined target regions. In allevaluated regions, mean regional SUVR_90–110_andcorresponding regional DVR were similar and showed Spearman’scorrelation ranging from 0.84 (insula) to 0.99 (entorhinal). In the CNgroups, mean regional SUVR_90–110_and DVR ranged from 0.59(amygdala) to 1.20 (lateral occipital) across target regions. In the ADgroup, the regional rank orders for SUVR_90–110_and DVRwere identical: Precuneus (SUVR_90–110_= 2.75)> Inferior Temporal > Rostral Middle Frontal > Fusiform> Lateral Occipital > Entorhinal > Precentral >Insula, Amygdala > Hippocampus (SUVR_90–110_=1.04).Figure 3Ashows violin plotsrepresenting baseline SUVR_90–110_for illustrative targetregions (entorhinal, amygdala, inferior temporal cortex). Individual Testand Retest SUVR_90–110_values for those same regions areshown inFigure 3C. In all evaluatedtarget regions, R_1_values were generally lower for AD (0.66(entorhinal) - 0.93 (insula)) than for oCN subjects (0.69 (entorhinal) -1.39 (insula)). The regional rank orders of R_1_values wereconsistent between the CN and AD groups: Insula > Precentral,Precuneus, Rostral Middle Frontal, Fusiform > Amygdala, Hippocampus> Inferior Temporal, Lateral Occipital > Entorhinal. Themeta-temporal composite region showed similar tau uptake and R_1_values as the inferior temporal region.

**Table 3.** [^18^F]MK-6240 target region outcome measures(SUVR_90–110_, DVR, R_1_) and theircorresponding T-RT (%).

	Non-PVC					
Region	SUVR _90–110_	SUVR _90–110_ T-RT (%)	DVR	DVR T-RT (%)	R _1_	R _1_ T-RT (%)
Entorhinal						
yCN	1.00 ± 0.53	N/A	0.95 ± 0.19	N/A	0.68 ± 0.07	N/A
oCN	1.11 ± 0.22	3.9 ± 4.5	1.04 ± 0.16	5.1 ± 3.2	0.69 ± 0.08	4.8 ± 4.4
AD	1.78 ± 0.26	3.9	1.72 ± 0.54	4.3	0.66 ± 0.03	1.5
Amygdala						
yCN	0.59 ± 0.13	N/A	0.69 ± 0.16	N/A	0.72 ± 0.07	N/A
oCN	0.76 ± 0.09	5.8 ± 2.8	0.85 ± 0.07	3.7 ± 1.9	0.91 ± 0.27	10.6 ± 16.4
AD	1.32 ± 0.47	2.4	1.29 ± 0.43	2.3	0.76 ± 0.05	1.6
Hippocampus						
yCN	0.64 ± 0.11	N/A	0.75 ± 0.13	N/A	0.79 ± 0.08	N/A
oCN	0.82 ± 0.12	6.3 ± 2.9	0.90 ± 0.09	3.8 ± 2.7	0.91 ± 0.25	11.3 ± 11.4
AD	1.04 ± 0.18	8.8	1.07 ± 0.17	9.2	0.77 ± 0.04	4.8
Fusiform						
yCN	0.98 ± 0.15	N/A	0.99 ± 0.13	N/A	0.95 ± 0.09	N/A
oCN	1.02 ± 0.12	8.6 ± 5.4	1.03 ± 0.08	5.8 ± 3.3	0.90 ± 0.06	5.2 ± 7.8
AD	2.22 ± 0.80	5.9	2.16 ± 0.84	9.0	0.84 ± 0.07	3.3
Inf. Temporal						
yCN	1.12 ± 0.18	N/A	1.06 ± 0.14	N/A	0.85 ± 0.10	N/A
oCN	1.08 ± 0.14	5.9 ± 4.3	1.05 ± 0.10	4.8 ± 3.1	0.83 ± 0.08	6.9 ± 3.8
AD	2.51 ± 0.98	3.2	2.51 ± 1.03	5.1	0.70 ± 0.06	6.3
Rostral Mid. Frontal						
yCN	0.97 ± 0.13	N/A	0.95 ± 0.10	N/A	1.02 ± 0.15	N/A
oCN	0.89 ± 0.11	6.2 ± 6.6	0.89 ± 0.09	4.6 ± 7.5	1.00 ± 0.12	6.9 ± 7.2
AD	2.25 ± 1.54	6.8	2.24 ± 1.49	2.6	0.83 ± 0.08	16.1
Lateral Occipital						
yCN	1.20 ± 0.12	N/A	1.12 ± 0.09	N/A	0.73 ± 0.09	N/A
oCN	1.15 ± 0.17	8.7 ± 4.9	1.08 ± 0.16	10.3 ± 6.0	0.81 ± 0.08	13.8 ± 6.0
AD	1.96 ± 0.80	7.6	1.97 ± 0.84	8.3	0.77 ± 0.06	1.0
Precuneus						
yCN	0.82 ± 0.15	N/A	0.87 ± 0.13	N/A	1.05 ± 0.12	N/A
oCN	0.86 ± 0.08	5.3 ± 2.9	0.91 ± 0.08	2.9 ± 2.1	1.06 ± 0.12	2.8 ± 2.9
AD	2.75 ± 1.36	2.9	2.77 ± 1.41	7.3	0.83 ± 0.04	5.7
Insula						
yCN	0.71 ± 0.11	N/A	0.81 ± 0.12	N/A	1.22 ± 0.26	N/A
oCN	0.78 ± 0.06	5.3 ± 3.0	0.86 ± 0.05	3.3 ± 2.0	1.39 ± 0.43	6.8 ± 5.4
AD	1.36 ± 0.52	1.0	1.32 ± 0.50	3.2	0.93 ± 0.17	5.4
Precentral						
yCN	0.88 ± 0.11	N/A	0.87 ± 0.08	N/A	0.92 ± 0.12	N/A
oCN	0.83 ± 0.08	5.8 ± 5.3	0.84 ± 0.06	3.9 ± 4.7	0.99 ± 0.12	8.3 ± 3.9
AD	1.56 ± 0.74	3.7	1.52 ± 0.67	4.4	0.86 ± 0.12	10.9
Meta-temporal						
yCN	1.02 ± 0.16	N/A	0.98 ± 0.13	N/A	0.86 ± 0.08	N/A
oCN	1.05 ± 0.12	4.1± 3.5	1.01 ± 0.08	3.9 ± 2.2	0.85 ± 0.05	7.0 ± 8.3
AD	2.23 ± 0.82	2.8	2.18 ± 0.83	1.1	0.67 ± 0.05	3.1
Extracerebral						
yCN	1.39 ± 0.34	N/A				
oCN	1.38 ± 0.55	12.1 ± 14.6	N/A	N/A	N/A	N/A
AD	1.33 ± 0.62	4.4				

All outcome measures were calculated using the CerGM as thereference region, and no PVC was applied. Baseline Test PETincludes 4 yCN, 10 oCN, and 3 AD subjects. Retest PET includes 7oCN and 1 AD subject. Values are expressed as mean ±standard deviation. The meta-temporal region is a composite ofentorhinal, parahippocampus, amygdala, fusiform, inferior, andmiddle temporal gyri.

#### 3.3.3 Target region SUVR _90–110_ , DVR, and R _1_ : T-RT and ICC (no PVC)

Table 3summarizesSUVR_90–110_, DVR, and R_1_T-RT valuesobtained using CerGM as the reference region for the examined targetregions. In the oCN group, target region SUVR_90–110_T-RTranged from 3.9% (entorhinal) to 8.7% (lateral occipital); DVR T-RT rangedfrom 2.9% (precuneus) to 10.3% (lateral occipital); and R_1_T-RTranged from 2.8% (precuneus) to 13.8% (lateral occipital). In the ADsubject, target region SUVR_90–110_T-RT ranged from 1.0%(insula) to 8.8% (hippocampus); DVR T-RT ranged from 2.3% (amygdala) to 9.2%(hippocampus); and R_1_T-RT ranged from 1.5% (entorhinal) to 16.1%(lateral occipital). The meta-temporal composite region showed T-RT for tauuptake and R_1_values similar (slightly lower) to the inferiortemporal region. Violin plots representing SUVR_90–110_T-RTfor illustrative target regions (entorhinal, amygdala, inferior temporalcortex) are shown inFigure 3B. The ICCvalues of SUVR_90–110_and DVR were moderate to excellent inall target regions (0.62 (fusiform)— 0.97 (entorhinal)), and ICC ofR_1_was moderate in the lateral occipital (0.52) and good toexcellent in all other regions, as shown inFigure 4. The Wilcoxon matched-pairs signed-rank test revealedno statistical differences between the Test and Retest scans forSUVR_90–110_, DVR, or R_1_in any of theevaluated target regions.

**Fig. 4.**
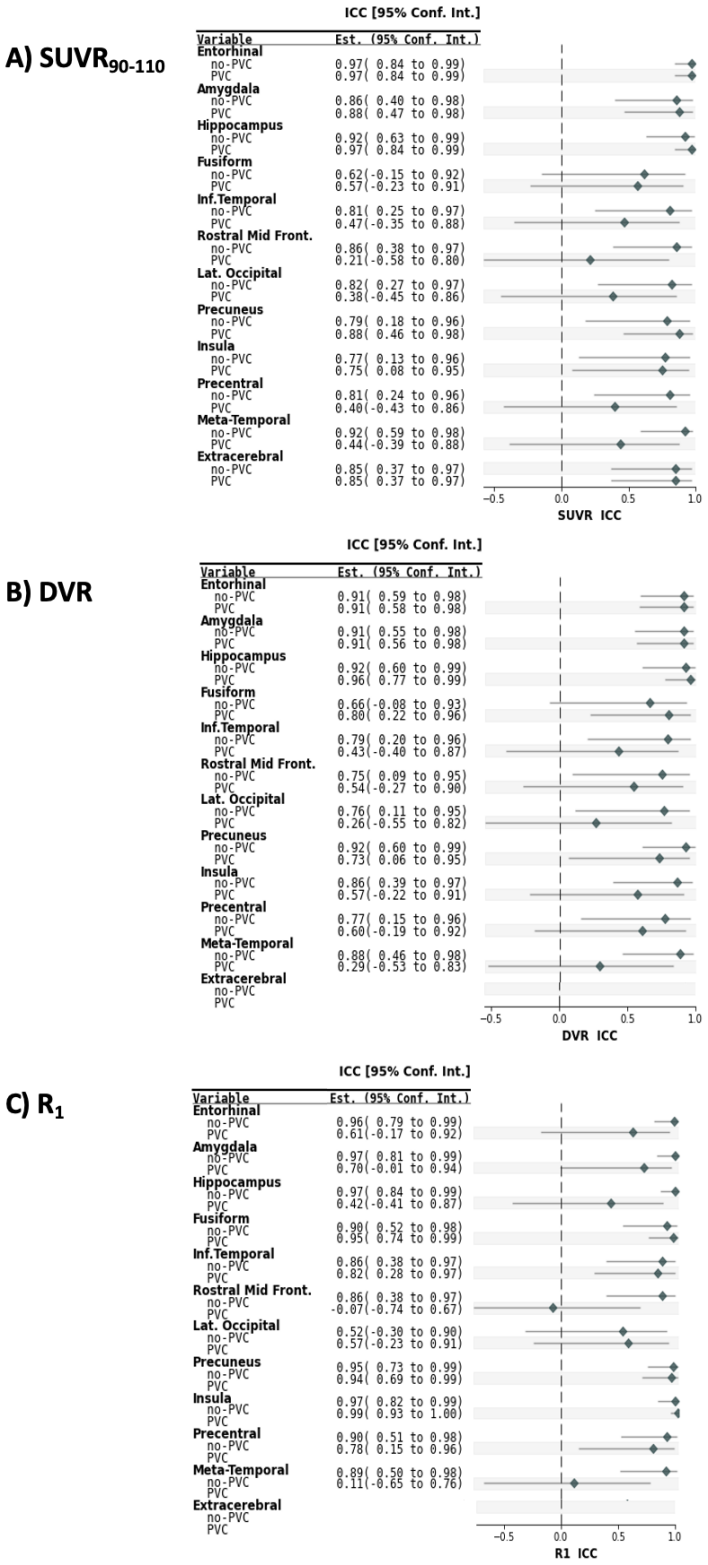
Intraclass correlation coefficients (ICC) and [95% confidenceinterval] for regional measures of: (A)SUVR_90–110_, (B) DVR, and (C) R_1_. ICCvalues provide a measure of the within-subject variability relativeto inter-subject variability and were calculated for the T-RT oCNgroup only (n = 7). The AD subject was excluded to avoid anincrease in the between-subjects variability explained by disease.No yCN subjects underwent Retest PET. In the extracerebral region,only SUVR_90–110_was evaluated. Regional ICC valueswere moderate to high (>0.6) in all target regions for allevaluated non-PVC outcome measures. PVC resulted in lower ICCs andlarger confidence intervals for regions such as the Precentral andInferior Temporal cortices, indicating high within-subjectvariability relative to the inter-subject variability.

#### 3.3.4 Target region: Impact of PVC on SUVR _90–110_ , DVR, and R _1_ T-RT

Supplementary Table3lists PVC SUVR_90–110_, PVC DVR, and PVCR_1_, and the corresponding T-RT values for all examined targetregions. The application of PVC resulted in a slight change in averageregional SUVR_90–110_and DVR (range: 0.29(amygdala)–1.33 (lateral occipital)) in the CN groups. In the ADgroup, PVC resulted in an increase in SUVR_90–110_and DVRin some cortical target regions (e.g.: fusiform and inferior temporal ~50%,precuneus ~70%), and the rank order of target region uptake was consistentwith that obtained with non-PVC. PVC R_1_values ranged from 0.70(entorhinal) to 1.70 (insula) and were, on average, ~20% higher than thenon-PVC R_1_values in both the CN and AD groups.

**Fig. 5.**
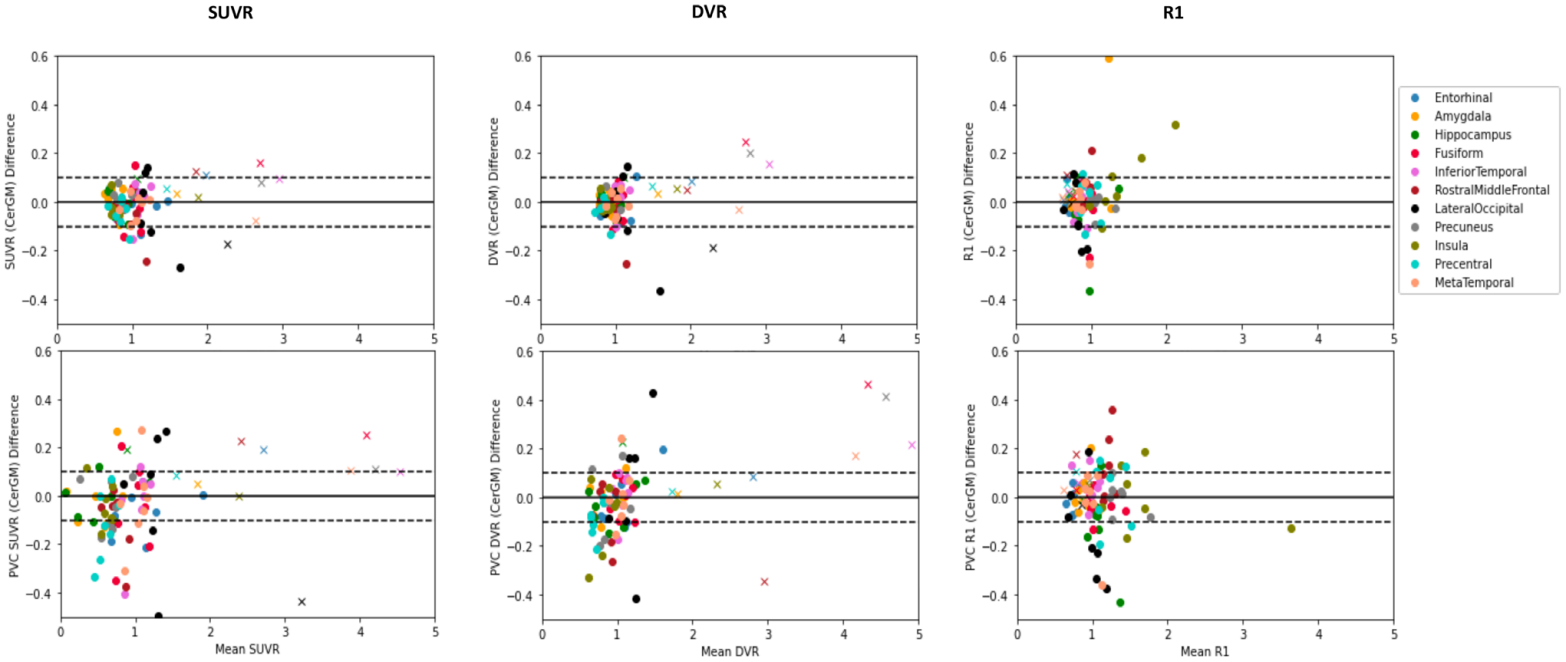
Bland–Altman plots for the regional outcome measures(SUVR_90–110_, DVR, and R_1_, withCerGM as reference), summarizing values across multiple targetregions. Results are shown for subjects with Test and Retest scans(7 oCN, represented with solid circles, and 1 AD, represented withcrosses), both with no PVC (top) and PVC applied (bottom). Thedashed line shows a difference of 0.1 for arbitrary reference.Differences between Test and Retest scans are similar forSUVR_90–110_, DVR, and R_1_measures.Applying PVC results in an increase in the Test and Retestdifferences and, thus, in the measurement variability.

The T-RT of PVC SUVR_90–110_and DVR increased by overtwofold compared with the respective non-PVC measures. In the oCN group,T-RT of PVC SUVR_90–110_ranged from 9.9% (entorhinal) to26.2% (precentral); T-RT of PVC DVR ranged from 6.3% (fusiform) to 21.8%(lateral occipital); and T-RT of PVC R_1_ranged from 3.5%(precuneus) to 19.9% (lateral occipital). For the AD subject, T-RT of PVCSUVR_90–110_ranged from 0 (insula) to 21.8%(hippocampus), T-RT of PVC DVR ranged from 1.5% (precentral) to 21.3%(hippocampus), and the T-RT of PVC R_1_ranged from 0.6%(entorhinal) to 22.6% (rostral middle frontal).SUVR_90–110_, DVR, and R_1_ICC values decreasednotably with PVC in some target regions (e.g., SUVR_90–110_Rostral Middle Frontal: 0.86 (non-PVC) to 0.21 (PVC),Fig. 4). The effect of PVC onSUVR_90–110_in selected target regions and on thecorresponding SUVR_90–110_T-RT is illustrated inFigure 3A and B, respectively, andindividual Test and Retest PVC SUVR_90–110_values are showninFigure 3D.Figure 5shows Bland–Altman plots summarizingvalues across multiple target regions for the examined outcome measureswithout and with the application of PVC (see Bland–Altman bias andlimit of agreement estimates by anatomical region inSupplementary Table4). Differences between Test and Retest inSUVR_90–110_, DVR, and R_1_values are similarand consistently small in all evaluated regions (generally < 0.1).PVC increases the differences between Test and Retest in all outcomemetrics, and thus the measurement variability.

### 3.4 Extracerebral region: SUV _90–110_ , SUVR _90–110_ , and corresponding T-RT

Tables 2and3summarize the SUV_90–110_,SUVR_90–110_, and the corresponding T-RT values for theextracerebral region. Extracerebral SUV_90–110_values in the CNgroups (yCN: 0.80; oCN: 0.77 g/mL) were higher than reference and target regionSUV_90–110_values (0.34–0.66 g/mL); AD group values(0.91 g/mL) were in the range of target region SUV_90–110_values (0.94–1.77 g/mL). Extracerebral SUVR_90–110_values were similar in the CN and AD groups (1.33–1.39) and higher thanthe target region SUVR_90–110_values in the CN groups(0.64–1.12). In the oCN group, the SUVR_90–110_T-RT wastwofold higher in the extracerebral region (~12%) than the average of examinedtarget regions (~6%), while in the AD subject, the SUVR_90–110_T-RT was similar to the average of examined target regions (~4%). Theextracerebral Test and Retest SUVR_90–110_displayed high inter-and intra-subject variability (Fig. 3C).The extracerebral T-RT variability increased slightly with the application ofPVC (~15% and 6% in the oCN and AD groups, respectively). ICC values were poorfor extracerebral SUV_90–110_(~0.4) but good forSUVR_90–110_(> 0.8), as summarized inFigure 4andSupplementary Figure2.

## 4 Discussion

In this study, we investigated the short-term (~ 3 weeks) T-RT characteristics ofdynamic [^18^F]MK-6240 outcomes in a group largely consisting ofcognitively normal older individuals using simultaneous PET/MRI. We reported T-RTresults for uptake in potential reference regions, for extracerebral off-targetsignal, as well as for estimates of tau burden and relative delivery indices intau-bearing target regions.

For the evaluated reference regions, we found acceptable T-RT variability inlate-frame SUV_90–110_measures, ranging from 6 to 13% across allsubjects. Currently, there is no consensus on the optimal reference region for[^18^F]MK-6240, and various studies have chosen different regions fornormalization (Fu et al., 2023;Gogola et al., 2020;Guehl et al., 2019;Lohith et al., 2019;Pascoal et al.,2018,2020b,^2021^;Salinas et al.,2019;Shuping et al., 2023). In arecent study,Fu et al. (2023)evaluatedlongitudinal changes in reference region SUV_90–110_, as well as theimpact of the reference region on target region outcomes. The authors suggestedthat, for cross-sectional studies, eroded CerGM may be preferred for differentiationbetween CN and AD groups, while eroded WM or pons may be preferred for detectinglongitudinal [^18^F]MK-6240 changes. Although theSUV_90–110_T-RT could influence the selection of the optimalreference region, comparative evaluations have not been reported. For oCN, ourresults showed the lowest SUV_90–110_T-RT variability in pons andCerGM, but no significant differences were observed between the evaluated regions.Since SUV_90–110_T-RT did not strongly favor a specific region, itshould be used as complementary but not as a main factor in reference regionselection. An important factor to consider is whether the chosen reference region isprone to spill-in contamination signal from high-uptake cortical regions andextracerebral regions.

In this work, we also assessed how the potential reference regions impactSUVR_90–110_T-RT in target regions. As expected, variousreference regions resulted in quantitative differences inSUVR_90–110_outcomes (lowest SUVR_90–110_values with CerGM or Inferior CerGM; highest with pons). Note that, as previouslyreported (Fu et al., 2023), WM andWM_4__mm_are prone to spill-in signal from the cortex in AD participants withhigh tau uptake, resulting in higher SUV_90–110_in the AD than inCN subjects. Therefore, WM and WM_4__mm_resulted in lower SUVR_90–110_in the AD than in theCN group, appearing not to be good reference regions in this study. For the set ofevaluated reference regions, target region SUVR_90–110_T-RT rangedfrom 2 to 15%, with no significant differences due to the choice of referenceregions. For the remaining analysis, we selected CerGM as the reference region, asit is one of the most used in the current literature (Bourgeat et al., 2023;Krishnadas et al., 2023;Vanderlinden etal., 2022).

For the evaluated target regions, we found low SUVR_90–110_T-RTvariability, with regional values ranging between 1 and 9% across all subjects, andan average SUVR_90–110_T-RT across all target regions and subjectsof 5%. These results are consistent with two previous reports, despite differencesin the study design and methodology. Using a standalone PET system,Salinas et al. (2019)determined SUVR using 90–120min of [^18^F]MK-6240 data (SUVR_90–120_) and the inferiorCerGM as reference region in a group consisting of 12 AD (65 ± 1 years) and 3CN (55 ± 7 years) subjects. With a similar T-RT interval (21 days), theyreported an average 6% target region T-RT across all subjects (ranging between 2 and9% in tau-rich and 2 and 21% in tau-poor regions). Using a PET/MRI system,Vanderlinden et al. (2022)reported long-term6-month [^18^F]MK-6240 SUVR_90–120_T-RT in a sampleconsisting of 10 CN (56 ± 12 years) using the CerGM as reference region. Formean whole grey matter, SUVR_90–120_T-RT was 2%, and regionalSUVR_90–120_T-RT ranged from 3 to 8%.

Our SUVR_90–110_T-RT results complement previous reports in two mainaspects: (1) The focus of our study was on older CN subjects (median age 69 years,IQR [66,71]), not well represented in previous studies that included younger CNsubjects (average age ~56 years). Our results thus fill an important gap inunderstanding the T-RT characteristics in oCN, a population especially important forlongitudinal observational studies involving largely these subjects (such as theHarvard Aging Brain Study (Dagley et al.,2017)). Second, the previous studies focused on the evaluation of T-RT inlarger (or aggregated) target regions but did not include important smaller regions,such as the entorhinal cortex or amygdala, that are known to be pathologicallyaffected in the early stages of AD. While using large ROIs has advantages in termsof lower sensitivity to image noise and contamination due to partial volume effects,the superiority of global or aggregate measures versus regional measurements has notbeen rigorously established for tau imaging. The anatomic distribution of[^18^F]MK-6240 retention, particularly in the early stages ofAD-related tauopathy, is variable within vulnerable areas and may be masked whenemploying large ROIs. Our data showed excellent SUVR_90–110_T-RTcharacteristics in important regions (e.g., 4% in the entorhinal cortex), suggesting[^18^F]MK-6240 is suitable for detecting early tau deposition as wellas for measuring longitudinal changes over time.

We also investigated differences between T-RT variability obtained from DVR withdynamic imaging and with late frame SUVR_90–110_in target regions.[^18^F]MK-6240 SUVR has been shown to lack a plateau in high taubinding areas (Guehl et al., 2019;Salinas et al., 2019), even 150 min afterinjection, which could introduce bias unless a precise acquisition time acrosslongitudinal assessments is maintained. This potential pitfall has also beendescribed for other tau tracers (Barret et al.,2017;Bullich et al., 2020;Kuwabara et al., 2018). Although our sampleconsists primarily of oCN with low tau accumulation, investigating differencesbetween DVR and SUVR T-RT may provide valuable information for the design ofprospective studies.Salinas et al. (2019)analyzed dynamic data and reported that the T-RT variability of V_T_andBP_ND_outcome measures was higher than the T-RT variability ofSUVR_90–120_across subjects. For the subset of CN subjects (n= 3), they reported an average T-RT of 22% for V_T_and 5% forSUVR_90–120_in tau-rich regions, whereas the T-RT ofBP_ND_exceeded 100% (likely due to the small number of CN subjects inthe study). Contrary to their results, we found low DVR variability, with targetregion DVR T-RT between 1 and 10% and similar to SUVR_90–110_T-RT(1–9%) across all subjects. When considering oCN only, DVR T-RT variabilitywas marginally but consistently lower than SUVR_90–110_T-RTvariability in all examined target regions except the entorhinal cortex and lateraloccipital. Given the potential bias of SUVR, good DVR reproducibility could favorthe use of this outcome measure in longitudinal studies aimed at detecting smallchanges in [^18^F]MK-6240 uptake.

In the present work, we also evaluated R_1_T-RT using dynamic data. Arecent study supports using the early phase of [^18^F]MK-6240 dynamic datato derive robust estimates of relative delivery R_1_as a quantitativeindex of relative cerebral perfusion (Guehl et al.,2023), but [^18^F]MK-6240 R_1_T-RT was still lacking.Our study demonstrated acceptable short-term R_1_T-RT variability in allevaluated target regions, ranging from 1 to 16% across all subjects, furthersupporting the viability of [^18^F]MK-6240 R_1_to robustlyevaluate longitudinal changes in perfusion.

We investigated the effects of an iterative voxel-based PVC on the evaluated outcomemeasurements. For oCN, PVC had a small effect on SUV_90–110_,SUVR_90–110_, and DVR, as these subjects present an overall lowand uniform uptake. For AD, PVC resulted in the expected patterns, with an increaseof signal in high tau-bearing regions (e.g., inferior temporal cortex), a decreaseof signal in areas prone to contamination (e.g., white matter), and no effect inregions not prone to contamination and with minimal tau accumulation (e.g., pons).The application of PVC increased T-RT variability for SUVR_90–110_,DVR, and R_1_in target regions, up to over twofold compared with the T-RTof non-PVC measures. These results are consistent with the increase in T-RTvariability in SUVR due to PVC observed byVanderlinden et al. (2022). Although they report smaller changes than inour study, this may be explained by the use of larger regions in their analyses,which are less affected by spill-in and spill-out effects. Due to the resultingincrease in inter-subject and T-RT variability in reference region and target regionuptake, caution should be taken when applying PVC to [^18^F]MK-6240 PETanalyses.

A large meta-temporal composite region was evaluated in this study. It showed similarmagnitudes of tau uptake (SUVR_90–110_and DVR) and R_1_values as the inferior temporal region, with slightly lower inter-subjectvariability. These observations provide additional support for the feasibility ofusing a meta-temporal composite as a reliable target to measure tau burden andrelative perfusion in clinical studies.

Contrary to common assumptions, our analysis revealed no clear relationship betweentarget region T-RT and ROI size. This is well illustrated by the entorhinal cortex,which, despite being the smallest region in this study (~650 voxels in the PETspace), exhibited one of the lowest T-RT values (3.9% TRT forSUVR_90–110_, 5.1% TRT for DVR, and 4.8% TRT for R_1_in oCN participants). This suggests that factors such as the anatomical location ofthe ROI (e.g., proximity to ventricles, extracerebral off-target signal, andhigh-tau ROIs) may have a larger impact on T-RT for [^18^F]MK-6240 outcomesthan ROI size alone, which has important implications for optimizing ROI selectionin clinical research.

A key part of this study is the evaluation of T-RT of [^18^F]MK-6240 uptakein the extracerebral space, which has been shown to present high signal in about 50%of subjects (Fu et al., 2023). Our findingsreveal notable heterogeneity in extracerebral uptake, consistent with recentliterature (Fu et al., 2023;McVea et al., 2024). We observed high inter-subjectvariability in both the location and magnitude of the extracerebral signal,particularly in the meningeal and sinus spaces. Some CN participants exhibited highmeningeal signal, while others showed relatively low signal. Even in subjects withhigh meningeal signal, the distribution was non-uniform. In CN participants, themeningeal uptake was generally more pronounced inferior to the cerebellum comparedwith the superior portion.

We also found considerable intra-subject T-RT variability in the extracerebralsignal. In the oCN group, T-RT variability for SUV_90–110_andSUVR_90–110_values was 10.9 ± 10.1% and 12.1 ±14.6%, respectively, with 57% of participants showing greater than 10% T-RTvariability in extracerebral SUV_90–110_. This aligns with recentresults fromMcVea et al. (2024), whoreported 44% of subjects showing >10% longitudinal variability in meningealsignal over an average 2.4-year follow-up.

Overall, our results indicate that extracerebral Test and RetestSUVR_90–110_present higher inter-subject and intra-subjectvariability than any of the evaluated target and reference regions, which couldaffect longitudinal target region quantification if not taken into account. AlthoughPVC is expected to reduce the effect of the extracerebral signal contamination intarget and reference regions, it is likely that some spill-in effects will remainafter its application. At the group level, the SUVR_90–110_T-RTvariability was slightly higher in the extracerebral region (8%) than in the averageof target regions (5%) and, when analyzing the oCN group separately, theSUVR_90–110_T-RT variability was two times higher in theextracerebral region (~12%) than in the average of examined target regions (~6%). InoCN, PVC increased the extracerebral SUVR_90–110_T-RT to 15%. In along-term T-RT evaluation of the extracerebral signal uptake,Vanderlinden et al. (2022)reported no significant meandifferences over a 6-month period. However, their data also display high individualvariability over time (15–35% for 4 of the 10 CN subjects). Due to thepotential of extracerebral signal to hamper accurate quantification, it is importantto examine the individual variability in addition to the group average. Ourobservations emphasize the importance of considering and potentially correcting forthe effects of extracerebral signal on [^18^F]MK-6240 PET analyses,especially in oCN.

One of the strengths of our study is the use of dynamic [^18^F]MK-6240 data.This allowed us to evaluate the T-RT characteristics of DVR and R_1_andcompare them with those of late-frame SUVR imaging. However, our study has somelimitations. The main limitation is the small sample size, and larger sample sizesmay be needed for robust validation of these results. In addition, our sample maynot be representative of older adults at high risk for AD, and future studies shouldinclude a larger proportion of amyloid-positive individuals. Furthermore, the lackof racial and ethnic diversity in the sample may limit the generalizability of thefindings to other populations. In addition, we did not have arterial data to measurethe input functions, and, therefore, our analyses were constrained to the evaluationof normalized outcome measures. Another limitation of our study is that we assumed auniform distribution of uptake inside the extracerebral mask, and the mask did notinclude the signal in the sinus space.

## 5 Conclusion

In low-signal oCN subjects, the T-RT variability of [^18^F]MK-6240 wasacceptable for SUV_90–110_in potential reference regions(6–11%), as well as in SUVR_90–110_(4–9%), DVR(3–10%), and R_1_(3–14%) in the evaluated target regions. Avoxel-based PVC resulted in increased T-RT variability forSUVR_90–110_(10–26%) and DVR (6–22%), but wassimilar for PVC R_1_(3–20%). Therefore, caution should be takenwhen applying PVC to [18F]MK-6240 tau and R_1_outcome measures.Extracerebral SUVR_90–110_exhibited higher T-RT variability (~12%)than other evaluated regions (~6%) and revealed higher intra-subject variabilitythat may affect the quantification in target and reference regions, particularly inlongitudinal studies. Our observations emphasize the importance of considering andpotentially correcting for the effects of extracerebral signal on[^18^F]MK-6240 PET analyses, especially in oCN. Our ﬁndings buildupon results from previous studies, further suggesting [^18^F]MK-6240 issuitable for detecting early tau deposition and measuring longitudinal changes overtime, as well as further supporting the viability of [^18^F]MK-6240R_1_to evaluate longitudinal changes in perfusion.

## Supplementary Material

Supplementary Material

## Data Availability

The data supporting this study’s findings are not openly available due toreasons of sensitivity and are available from the corresponding author uponreasonable request. Data are in controlled access data storage at MassachusettsGeneral Hospital.
