# Supplementary material for: Test–retest performance of [ 18 F]MK-6240 tau burden and relative delivery indices in cognitively normal older subjects using PET/MRI
Source: Imaging Neurosci (Camb). 2024 Dec 20;2:imag-2-00402. doi: 10.1162/imag_a_00402 (PMC12315748; doi:10.1162/imag_a_00402)
Supplement: Supplementary Material [file imag_a_00402-supp.pdf]

*Supplementary Table 1: Baseline* $SUV_{90\text{-}110}$*, corrected for partial volume (PVC) with the iterative Yang method (for 4 yCN, 10 oCN, and 3 AD subject), and corresponding* $SUV_{90\text{-}110}$ *T-RT(%). Retest PET includes 7 oCN and one AD subject (no yCN subjects underwent Retest PET). Values are expressed as mean±standard deviation. PVC was not performed for the eroded reference regions (i.e., CerGM3mm and WM4mm) as erosion constitutes a different approach to eliminate spill-in signal in those regions.*

| Region | PVC iterative Yang | | | | |
|---|---|---|---|---|---|
| | SUV (g/mL yCN, n=4) | SUV (g/mL oCN, n=10) | SUV T-RT (%, oCN, n=7) | SUV (g/mL AD, n=3) | SUV T-RT (%, AD, n=1) |
| CerGM | 0.59±0.19 | 0.58±0.16 | 9.6±7.2 | 0.68±0.15 | 11.5 |
| CerGM3mm | N/A | N/A | N/A | N/A | N/A |
| Inferior CerGM | 0.59±0.19 | 0.59±0.16 | 12.0±8.4 | 0.64±0.14 | 9.1 |
| WholeCer | 0.54±0.17 | 0.55±0.16 | 9.6±6.1 | 0.61±0.14 | 11.9 |
| CerWM | 0.32±0.08 | 0.42±0.13 | 13.7±11.0 | 0.39±0.12 | 13.1 |
| WM | 0.38±0.08 | 0.46±0.11 | 8.0±5.1 | 0.92±0.36 | 9.45 |
| WM4mm | N/A | N/A | N/A | N/A | N/A |
| Pons | 0.31±0.07 | 0.39±0.11 | 7.2±4.7 | 0.37±0.06 | 12.9 |
| Entorhinal | 0.54±0.09 | 0.68±0.33 | 16.2±15.9 | 1.48±0.55 | 4.3 |
| Amygdala | 0.17±0.09 | 0.32±0.18 | 23.1±23.1 | 0.88±0.45 | 8.8 |
| Hippocampus | 0.19±0.09 | 0.37±0.24 | 20.2±20.3 | 0.60±0.17 | 10.5 |
| Fusiform | 0.54±0.14 | 0.60±0.22 | 18.5±22.1 | 2.11±0.95 | 5.3 |
| Inf. Temporal | 0.65±0.14 | 0.63±0.22 | 17.2±22.1 | 2.39±1.04 | 9.2 |
| Precuneus | 0.40±0.15 | 0.43±0.19 | 19.2±16.0 | 2.86±1.32 | 8.8 |
| Rostral Mid. Frontal | 0.50±0.19 | 0.41±0.15 | 25.9±29.0 | 1.99±1.40 | 2.0 |
| Lateral Occipital | 0.77±0.24 | 0.70±0.20 | 27.0±19.6 | 1.69±0.70 | 24.8 |
| Insula | 0.32±0.11 | 0.37±0.13 | 15.5±16.7 | 0.94±0.71 | 11.5 |
| Precentral | 0.44±0.18 | 0.35±0.15 | 32.5±31.5 | 1.12±0.50 | 5.8 |
| Meta-temporal | 0.57±0.13 | 0.63±0.20 | 21.1±19.8 | 2.09±0.86 | 8.7 |
| Extracerebral | 0.97±0.13 | 0.93±0.15 | 11.4±11.1 | 1.04±0.29 | 17.8 |

*Supplementary Table 2:* Effect of normalization by different reference regions in $SUVR_{90\text{-}110}$ and $SUVR_{90\text{-}110}$ T-RT.

| **Region** | **SUVR CerGM** | **T-RT (%)** | **SUVR $CerGM_{3mm}$** | **T-RT (%)** | **SUVR InfCerGM** | **T-RT (%)** | **SUVR WholeCer** | **T-RT (%)** | **SUVR CerWM** | **T-RT (%)** | **SUVR WM** | **T-RT (%)** | **SUVR $WM_{4mm}$** | **T-RT (%)** | **SUVR Pons** | **T-RT (%)** |
|---|---|---|---|---|---|---|---|---|---|---|---|---|---|---|---|---|
| Entorhinal | | | | | | | | | | | | | | | | |
| yCN | 1.00±0.53 | N/A | 1.19±0.44 | N/A | 0.97±0.24 | N/A | 1.08±0.29 | N/A | 1.55±0.45 | N/A | 1.41±0.32 | N/A | 1.66±0.41 | N/A | 1.86±0.38 | N/A |
| oCN | 1.11±0.22 | 3.9±4.5 | 1.25±0.31 | 5.6±5.6 | 1.10±0.19 | 3.9±4.5 | 1.17±0.24 | 4.8±4.7 | 1.50±0.43 | 10.8±8.5 | 1.41±0.27 | 7.1±4.7 | 1.55±0.36 | 10.0±5.9 | 1.75±0.43 | 9.6±3.1 |
| AD | 1.78±0.26 | 3.9 | 2.28±0.93 | 2.4 | 1.93 ±0.72 | 5.6 | 1.94 ±0.61 | 5.8 | 2.86 ±1.14 | 5.9 | 1.16 ±0.12 | 2.5 | 1.65 ±0.22 | 2.9 | 3.34 ±0.91 | 6.8 |
| Amygdala | | | | | | | | | | | | | | | | |
| yCN | 0.59±0.13 | N/A | 0.70±0.21 | N/A | 0.58±0.13 | N/A | 0.63±0.14 | N/A | 0.91±0.20 | N/A | 0.81±0.07 | N/A | 0.95±0.03 | N/A | 1.08±0.09 | N/A |
| oCN | 0.76±0.09 | 5.8±2.8 | 0.85±0.12 | 5.5±3.5 | 0.76±0.09 | 5.8±4.5 | 0.80±0.09 | 5.2±3.2 | 1.02±0.16 | 7.9±6.1 | 0.97±0.10 | 6.3±4.2 | 1.06±0.14 | 6.8±4.7 | 1.19±0.14 | 4.6±6.2 |
| AD | 1.32±0.47 | 2.4 | 1.71±0.78 | 0.8 | 1.47±0.65 | 2.4 | 1.44±0.53 | 2.6 | 2.14±0.94 | 2.7 | 0.85±0.03 | 0.7 | 1.21±0.17 | 0.3 | 2.48±0.83 | 3.5 |
| Hippocampus | | | | | | | | | | | | | | | | |
| yCN | 0.64±0.11 | N/A | 0.75±0.19 | N/A | 0.62±0.11 | N/A | 0.68±0.12 | N/A | 0.98±0.18 | N/A | 0.89±0.06 | N/A | 1.04±0.10 | N/A | 1.18±0.05 | N/A |
| oCN | 0.82±0.12 | 6.3±2.9 | 0.92±0.15 | 5.0±3.3 | 0.82±0.12 | 6.3±4.5 | 0.86±0.12 | 5.6±2.6 | 1.10±0.18 | 6.3±4.5 | 1.05±0.14 | 5.9±4.1 | 1.14±0.20 | 5.2±4.7 | 1.28±0.19 | 5.2±5.2 |
| AD | 1.04±0.18 | 8.8 | 1.32±0.39 | 5.6 | 1.10±0.27 | 8.8 | 1.13±0.21 | 9.1 | 1.66±0.47 | 9.1 | 0.71±0.19 | 5.7 | 0.99±0.19 | 6.2 | 1.96±0.27 | 10.0 |
| Fusiform | | | | | | | | | | | | | | | | |
| yCN | 0.98±0.15 | N/A | 1.16±0.28 | N/A | 0.96±0.15 | N/A | 1.06±0.17 | N/A | 1.52±0.25 | N/A | 1.38±0.11 | N/A | 1.62±0.20 | N/A | 1.82±0.09 | N/A |
| oCN | 1.02±0.12 | 8.6±5.4 | 1.14±0.19 | 10.5±6.6 | 1.01±0.11 | 8.6±5.4 | 1.08±0.14 | 9.2±5.7 | 1.37±0.26 | 14.5±9.0 | 1.30±0.14 | 10.4±4.0 | 1.42±0.21 | 11.7±4.9 | 1.60±0.24 | 11.0±4.4 |
| AD | 2.22±0.80 | 5.9 | 2.86±1.32 | 2.8 | 2.37±1.04 | 5.9 | 2.42±0.90 | 6.2 | 3.58±1.56 | 6.3 | 1.43±0.09 | 2.8 | 2.02±0.24 | 3.3 | 4.17±1.41 | 7.1 |
| Inf. Temporal | | | | | | | | | | | | | | | | |
| yCN | 1.12±0.18 | N/A | 1.32±0.34 | N/A | 1.09±0.17 | N/A | 1.20±0.20 | N/A | 1.73±0.31 | N/A | 1.58±0.20 | N/A | 1.86±0.32 | N/A | 2.09±0.22 | N/A |
| oCN | 1.08±0.14 | 5.9±4.3 | 1.21±0.23 | 8.0±4.8 | 1.07±0.12 | 5.9±4.3 | 1.14±0.16 | 6.8±4.5 | 1.46±0.32 | 12.4±8.9 | 1.38±0.15 | 8.3±4.1 | 1.50±0.20 | 10.7±5.5 | 1.70±0.28 | 10.3±5.6 |
| AD | 2.51±0.98 | 3.2 | 3.24±1.57 | 0.0 | 2.72±1.22 | 3.2 | 2.74±1.11 | 3.4 | 4.07±1.93 | 3.5 | 1.59±0.04 | 0.0 | 2.27±0.04 | 0.5 | 4.70±1.72 | 4.4 |
| Rostr Mid Front | | | | | | | | | | | | | | | | |
| yCN | 0.97±0.13 | N/A | 1.14±0.20 | N/A | 0.95±0.14 | N/A | 1.05±0.13 | N/A | 1.50±0.14 | N/A | 1.37±0.10 | N/A | 1.62±0.26 | N/A | 1.82±0.16 | N/A |
| oCN | 0.89±0.11 | 6.2±6.6 | 1.01±0.22 | 6.9±6.2 | 0.89±0.10 | 6.2±6.6 | 0.94±0.13 | 6.3±6.8 | 1.21±0.30 | 10.1±9.9 | 1.14±0.16 | 5.7±6.9 | 1.24±0.19 | 8.6±9.7 | 1.41±0.27 | 8.2±9.5 |
| AD | 2.25±1.54 | 6.8 | 2.93±2.17 | 3.6 | 2.39±1.49 | 6.8 | 2.46±1.71 | 7.0 | 3.74±2.92 | 7.1 | 1.35±0.57 | 3.7 | 2.03±1.25 | 4.1 | 4.18±2.72 | 8.0 |
| Lat. Occipital | | | | | | | | | | | | | | | | |
| yCN | 1.20±0.12 | N/A | 1.41±0.28 | N/A | 1.17±0.10 | N/A | 1.29±0.14 | N/A | 1.86±0.25 | N/A | 1.71±0.30 | N/A | 1.78±0.46 | N/A | 2.26±0.35 | N/A |
| oCN | 1.15±0.17 | 8.7±4.9 | 1.30±0.30 | 9.6±4.1 | 1.14±0.14 | 8.7±4.9 | 1.21±0.19 | 9.2±5.2 | 1.57±0.42 | 15.3±6.4 | 1.48±0.25 | 10.6±6.6 | 1.61±0.31 | 14.5±8.2 | 1.82±0.39 | 14.2±8.4 |
| AD | 1.96±0.80 | 7.6 | 2.53±1.25 | 10.7 | 2.12±0.94 | 7.6 | 2.14±0.90 | 7.3 | 3.18±1.60 | 7.3 | 1.24±0.09 | 10.7 | 2.02±0.51 | 10.3 | 3.66±1.38 | 6.3 |
| Precuneus | | | | | | | | | | | | | | | | |
| yCN | 0.82±0.15 | N/A | 0.97±0.23 | N/A | 0.81±0.16 | N/A | 0.89±0.16 | N/A | 1.27±0.20 | N/A | 1.15±0.03 | N/A | 1.35±0.11 | N/A | 1.53±0.09 | N/A |
| oCN | 0.86±0.08 | 5.3±2.9 | 0.96±0.12 | 4.9±3.8 | 0.86±0.09 | 5.3±2.9 | 0.91±0.09 | 4.3±3.2 | 1.15±0.16 | 5.8±5.3 | 1.10±0.04 | 3.2±3.3 | 1.19±0.08 | 2.3±1.6 | 1.35±0.12 | 2.6±1.2 |
| AD | 2.75±1.36 | 2.9 | 3.55±2.02 | 0.2 | 2.87±1.34 | 2.9 | 3.00±1.52 | 3.2 | 4.50±2.68 | 3.2 | 1.71±0.34 | 0.2 | 2.50±0.97 | 0.2 | 5.12±2.36 | 4.1 |
| Insula | | | | | | | | | | | | | | | | |
| yCN | 0.71±0.11 | N/A | 0.83±0.17 | N/A | 0.69±0.12 | N/A | 0.76±0.11 | N/A | 1.09±0.14 | N/A | 0.99±0.03 | N/A | 1.16±0.13 | N/A | 1.31±0.07 | N/A |
| oCN | 0.78±0.06 | 5.3±3.0 | 0.87±0.09 | 6.5±3.4 | 0.77±0.07 | 5.3±3.0 | 0.82±0.06 | 5.5±2.4 | 1.04±0.13 | 7.8±7.3 | 0.99±0.03 | 3.4±3.4 | 1.08±0.09 | 3.6±3.2 | 1.21±0.09 | 2.9±2.7 |
| AD | 1.36±0.52 | 1.0 | 1.75±0.85 | 2.1 | 1.51±0.77 | 1.0 | 1.48±0.58 | 1.3 | 2.17±0.93 | 1.4 | 0.88±0.17 | 2.1 | 1.23±0.09 | 1.6 | 2.55±0.95 | 2.2 |
| Precentral | | | | | | | | | | | | | | | | |
| yCN | 0.88±0.11 | N/A | 1.03±0.16 | N/A | 0.86±0.13 | N/A | 0.95±0.11 | N/A | 1.36±0.10 | N/A | 1.24±0.13 | N/A | 1.47±0.28 | N/A | 1.65±0.19 | N/A |
| oCN | 0.83±0.08 | 5.8±5.3 | 0.93±0.15 | 6.0±4.4 | 0.82±0.08 | 5.8±5.3 | 0.87±0.09 | 5.8±5.1 | 1.11±0.20 | 8.0±8.4 | 1.05±0.08 | 4.8±5.0 | 1.15±0.11 | 6.2±8.5 | 1.29±0.16 | 6.3±8.3 |
| AD | 1.56±0.74 | 3.7 | 2.01±1.11 | 0.5 | 1.61±0.69 | 3.7 | 1.70±0.83 | 3.9 | 2.55±1.48 | 4.0 | 0.98±0.20 | 0.5 | 1.43±0.54 | 1.0 | 2.91±1.27 | 4.9 |
| Meta-temporal | | | | | | | | | | | | | | | | |
| yCN | 1.02±0.16 | N/A | 1.20±0.29 | N/A | 0.99±0.15 | N/A | 1.09±0.17 | N/A | 1.57±0.26 | N/A | 1.43±0.14 | N/A | 1.89±0.32 | N/A | 1.89±0.14 | N/A |
| oCN | 1.05±0.12 | 4.1± 3.5 | 1.17±0.22 | 6.2±3.9 | 1.04±0.10 | 4.1±3.5 | 1.10±0.14 | 5.0±3.6 | 1.41±0.30 | 10.8±8.1 | 1.33±0.13 | 6.4±3.4 | 1.47±0.32 | 10.0±3.4 | 1.64±0.26 | 9.5±2.8 |
| AD | 2.23±0.82 | 2.8 | 2.90±1.35 | 4.6 | 2.44±1.05 | 2.8 | 2.44±0.92 | 2.6 | 3.60±1.62 | 2.7 | 1.42±0.05 | 1.1 | 1.69±0.64 | 0.7 | 4.11±1.36 | 0.1 |
| Extracerebral | | | | | | | | | | | | | | | | |
| yCN | 1.39±0.34 | N/A | 1.66±0.57 | N/A | 1.36±0.31 | N/A | 1.45±0.69 | N/A | 2.16±0.57 | N/A | 1.97±0.45 | N/A | 2.32±0.62 | N/A | 2.61±0.57 | N/A |
| oCN | 1.38±0.55 | 12.1±14.6 | 1.59±0.81 | 13.8±13.9 | 1.37±0.52 | 12.1±14.6 | 1.46±0.61 | 12.7±14.1 | 1.93±1.07 | 16.0±12.9 | 1.78±0.75 | 15.5±15.1 | 1.93±0.80 | 16.4±14.3 | 2.21±1.07 | 14.8±12.5 |
| AD | 1.33±0.62 | 4.4 | 1.69±0.91 | 7.6 | 1.40±0.59 | 4.4 | 1.50±0.38 | 4.2 | 2.16±1.26 | 4.1 | 0.89±0.33 | 7.6 | 1.28±0.57 | 7.1 | 2.49±1.06 | 3.2 |

*Supplementary Table 3: Target region PVC outcome measures ($SUVR_{90\text{-}110}$, DVR, $R_1$) and their corresponding T-RT (%). All outcomes were calculated using CerGM as reference and corrected for partial volume effects using the Iterative Yang method. Not all subjects underwent Retest PET (only one AD subject and none of the yCN; consequently, T-RT data is unavailable for that group). Values are expressed as mean±standard deviation.*

| Region | PVC iterative Yang | | | | | |
|---|---|---|---|---|---|---|
| | SUVR | SUVR T-RT (%) | DVR | DVR T-RT (%) | $R_1$ | $R_1$ T-RT (%) |
| **Entorhinal** | | | | | | |
| yCN | 0.99±0.36 | N/A | 0.94±0.28 | N/A | 0.74±0.09 | N/A |
| oCN | 1.16±0.40 | 9.9±9.7 | 1.12±0.31 | 8.5±3.6 | 0.70±0.11 | 6.6±3.1 |
| AD | 2.27±0.88 | 7.1 | 2.17±0.88 | 3.2 | 0.71±0.02 | 0.6 |
| **Amygdala** | | | | | | |
| yCN | 0.29±0.13 | N/A | 0.64±0.17 | N/A | 0.74±0.14 | N/A |
| oCN | 0.51±0.25 | 21.5±16.8 | 0.84±0.20 | 8.0±5.0 | 0.92±0.16 | 7.2±5.8 |
| AD | 1.33±0.62 | 2.6 | 1.30±0.53 | 0.9 | 0.87±0.04 | 0.9 |
| **Hippocampus** | | | | | | |
| yCN | 0.33±0.12 | N/A | 0.63±0.16 | N/A | 0.94±0.11 | N/A |
| oCN | 0.56±0.31 | 18.5±12.7 | 0.93±0.25 | 8.3±4.7 | 1.08±0.16 | 13.0±8.9 |
| AD | 0.88±0.09 | 21.8 | 1.08±0.11 | 21.3 | 0.94±0.11 | 0.9 |
| **Fusiform** | | | | | | |
| yCN | 0.96±0.21 | N/A | 1.04±0.15 | N/A | 1.03±0.08 | N/A |
| oCN | 1.01±0.22 | 17.5±14.5 | 1.10±0.11 | 6.3±3.3 | 1.05±0.15 | 4.7±3.8 |
| AD | 3.24±1.46 | 6.2 | 3.20±1.58 | 10.8 | 0.94±0.14 | 3.9 |
| **Inf. Temporal** | | | | | | |
| yCN | 1.15±0.22 | N/A | 1.10±0.19 | N/A | 1.01±0.14 | N/A |
| oCN | 1.07±0.21 | 10.8±15.8 | 1.10±0.11 | 7.1±5.0 | 0.94±0.13 | 8.0±6.2 |
| AD | 3.71±1.77 | 2.3 | 3.84±1.91 | 4.5 | 0.80±0.08 | 7.6 |
| **Rostral Mid. Frontal** | | | | | | |
| yCN | 0.85±0.22 | N/A | 0.93±0.15 | N/A | 1.30±0.19 | N/A |
| oCN | 0.68±0.12 | 20.6±21.7 | 0.81±0.13 | 10.4±9.8 | 1.31±0.20 | 10.1±10.2 |
| AD | 3.41±3.10 | 9.4 | 3.41±2.91 | 11.6 | 1.03±0.15 | 22.6 |
| **Lateral Occipital** | | | | | | |
| yCN | 1.33±0.13 | N/A | 1.33±0.19 | N/A | 0.77±0.13 | N/A |
| oCN | 1.21±0.20 | 19.9±13.1 | 1.18±0.23 | 21.8±13.5 | 0.95±0.17 | 19.9±10.2 |
| AD | 2.68±1.40 | 13.4 | 2.80±1.54 | 17.3 | 0.91±0.13 | 3.6 |
| **Precuneus** | | | | | | |
| yCN | 0.71±0.22 | N/A | 0.85±0.19 | N/A | 1.28±0.15 | N/A |
| oCN | 0.69±0.21 | 17.1±9.6 | 0.91±0.08 | 12.7±9.2 | 1.37±0.18 | 3.5±2.9 |
| AD | 4.68±3.10 | 2.6 | 4.92±3.30 | 9.1 | 1.06±0.09 | 7.1 |
| **Insula** | | | | | | |
| yCN | 0.56±0.13 | N/A | 0.87±0.25 | N/A | 1.34±0.23 | N/A |
| oCN | 0.61±0.11 | 15.6±10.9 | 0.81±0.16 | 15.1±19.0 | 1.70±0.74 | 6.5±3.8 |
| AD | 1.37±0.89 | 0.0 | 1.38±0.84 | 2.4 | 1.05±0.10 | 5.1 |
| **Precentral** | | | | | | |
| yCN | 0.74±0.20 | N/A | 0.83±0.13 | N/A | 1.10±0.18 | N/A |
| oCN | 0.58±0.16 | 26.2±25.8 | 0.73±0.16 | 13.3±17.2 | 1.27±0.20 | 9.9±4.3 |
| AD | 1.83±1.22 | 5.6 | 1.77±0.99 | 1.5 | 1.12±0.25 | 13.8 |
| **Meta-temporal** | | | | | | |
| yCN | 1.00±0.18 | N/A | 0.99±0.16 | N/A | 0.99±0.09 | N/A |
| oCN | 1.05±0.18 | 21.5±16.8 | 1.06±0.11 | 8.1±8.0 | 0.97±0.07 | 8.8±13.9 |
| AD | 3.25±1.49 | 2.6 | 3.26±1.58 | 4.1 | 0.73±0.08 | 5.0 |
| **Extracerebral** | | | | | | |
| yCN | 1.77±0.57 | N/A | | | | |
| oCN | 1.82±0.95 | 15.1±18.6 | N/A | N/A | N/A | N/A |
| AD | 1.66±0.90 | 6.3 | | | | |

*Supplementary Figure 1: Test (T) and Retest (RT) [$^{18}$F]MK-6240 SUVR images (using cerebellar grey matter as the reference region) overlaid on simultaneously acquired structural MRIs for all participants (7 oCN, 1AD). Extracerebral uptake was observed in the meninges and sinuses, with high inter-subject variability and, for some participants, also high intra-subject variability (e.g., Subject 7).*

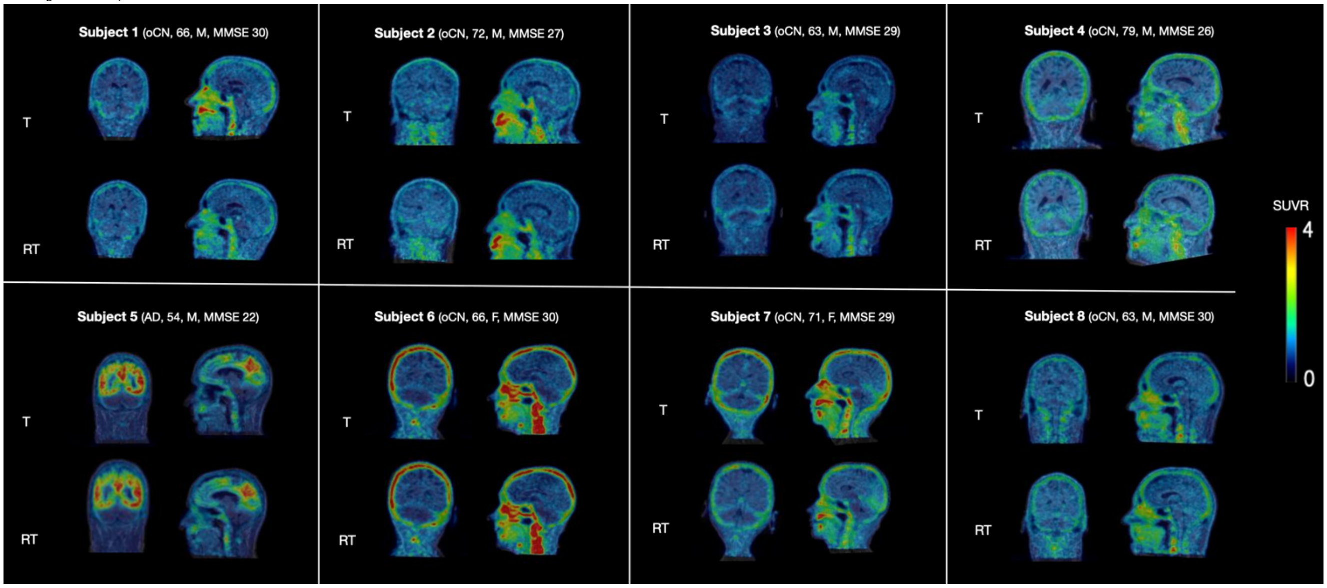


*Supplementary Figure 2: Intraclass correlation coefficients (ICC) and their corresponding [95% confidence interval] for regional $SUV_{90-110}$ in the reference (left) and target (right) regions, with and without PVC. The ICC values were calculated for each ROI across oCN subjects who underwent the Retest scans (n=7). The AD subject was excluded from the ICC calculation to avoid an increase in variability explained by disease; no yCN subjects underwent Retest PET.*

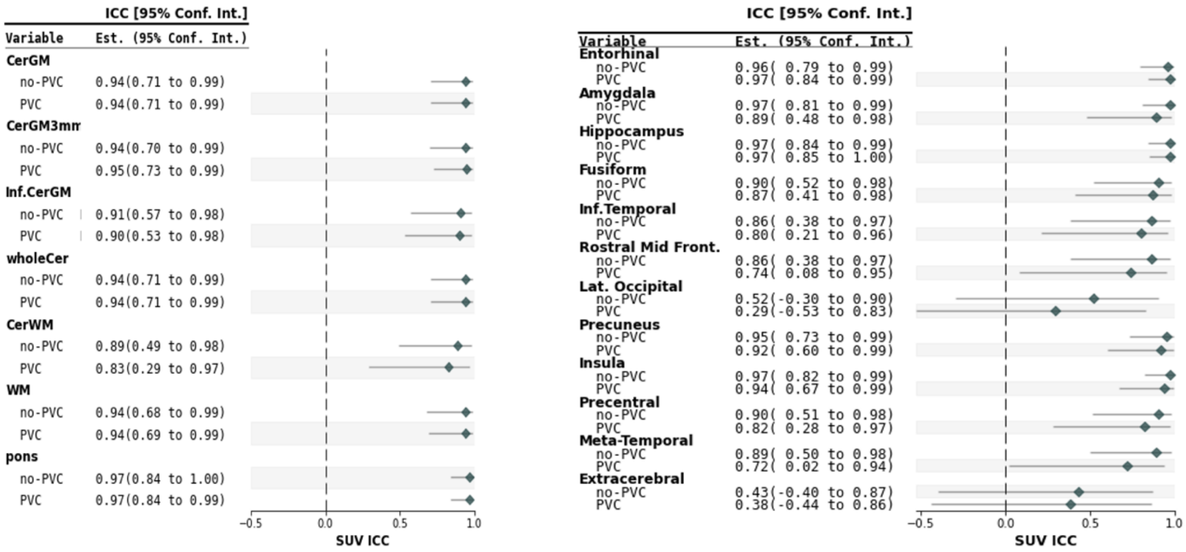

*Supplementary Table 4: Bland-Altman bias estimates and limits of agreement (LOA) for test-retest variability across multiple brain regions, both with and without partial volume correction (PVC), for different tau PET imaging outcomes (SUVR, DVR, R1). The table includes bias estimates (mean differences between test and retest measurements) and the corresponding lower and upper LOA values, which represent the range within which 95% of the differences between test-retest measurements are expected to fall. The analysis is performed both including all subjects ("all subjects") and excluding the AD subject ("no AD subject") to assess the impact of higher tau burden on measurement variability.*

| SUVR (non-PVC) | | | | | | |
|---|---|---|---|---|---|---|
| **Region** | **Bias (all subjects)** | **Lower LOA (all subjects)** | **Upper LOA (all subjects)** | **Bias (no AD subject)** | **Lower LOA (no AD subject)** | **Upper LOA (no AD subject)** |
| Entorhinal | 0.007 (-0.053, 0.068) | -0.134 (-0.242, -0.026) | 0.149 (0.041, 0.257) | 0.024 (-0.030, 0.078) | -0.091 (-0.189, 0.007) | 0.139 (0.041, 0.237) |
| Amygdala | -0.005 (-0.049, 0.039) | -0.108 (-0.186, -0.029) | 0.098 (0.019, 0.176) | -0.000 (-0.051, 0.050) | -0.108 ( -0.199, -0.017) | 0.107 (0.016, 0.198) |
| Hippocampus | -0.017 (-0.070, 0.036) | -0.141 (-0.235, -0.046) | 0.107 (0.012, 0.201) | -0.006 (-0.061, 0.049) | -0.122 (-0.221, -0.024) | 0.110 (0.011, 0.209) |
| Fusiform | -0.004 (-0.101, 0.092) | -0.230 (-0.402, -0.058) | 0.221 (0.049, 0.393) | 0.018 (-0.079, 0.114) | -0.186 (-0.360, -0.013) | 0.222 (0.049, 0.396) |
| Inferior Temporal | -0.009 (-0.077, 0.060) | -0.170 (-0.293, -0.047) | 0.153 (0.030, 0.275) | 0.003 (-0.071, 0.078) | -0.155 (-0.289, -0.020) | 0.162 (0.027, 0.296) |
| Rostral Middle Frontal | 0.041 (-0.044, 0.126) | -0.159 (-0.311, -0.007) | 0.240 (0.088, 0.393) | 0.065 (-0.012, 0.141) | -0.098 (-0.236, 0.040) | 0.227 (0.089, 0.365) |
| Lateral Occipital | 0.042 (-0.079, 0.163) | -0.242 (-0.459, -0.026) | 0.326 (0.109, 0.542) | 0.023 (-0.112, 0.158) | -0.263 (-0.506, -0.020) | 0.309 (0.066, 0.552) |
| Precuneus | -0.009 (-0.057, 0.039) | -0.121 (-0.207, -0.036) | 0.103 (0.018, 0.188) | 0.001 (-0.049, 0.051) | -0.104 (-0.193, -0.015) | 0.106 (0.017, 0.195) |
| Insula | 0.013 (-0.024, 0.051) | -0.074 (-0.141, -0.007) | 0.101 (0.034, 0.168) | 0.018 (-0.025, 0.061) | -0.072 (-0.149, 0.004) | 0.108 (0.032, 0.185) |
| Precentral | 0.032 (-0.022, 0.086) | -0.096 (-0.193, 0.002) | 0.159 (0.062, 0.256) | 0.044 (-0.011, 0.099) | -0.073 (-0.172, 0.026) | 0.161 (0.062, 0.260) |
| Meta Temporal | 0.025 (-0.019, 0.069) | -0.078 (-0.157, 0.001) | 0.129 (0.050, 0.208) | 0.018 (-0.031, 0.067) | -0.085 (-0.173, 0.003) | 0.122 (0.034, 0.210) |
| **SUVR (PVC)** | | | | | | |
| **Region** | **Bias (all subjects)** | **Lower LOA (all subjects)** | **Upper LOA (all subjects)** | **Bias (no AD subject)** | **Lower LOA (no AD subject)** | **Upper LOA (no AD subject)** |
| Entorhinal | 0.037 (-0.073, 0.147) | -0.222 (-0.419, -0.024) | 0.296 (0.099, 0.493) | 0.070 (-0.024, 0.164) | -0.129 (-0.298, 0.040) | 0.269 (0.100, 0.437) |
| Amygdala | 0.001 (-0.106, 0.108) | -0.250 (-0.442, -0.059) | 0.252 (0.061, 0.444) | 0.008 (-0.118, 0.135) | -0.260 (-0.488, -0.032) | 0.276 (0.048, 0.504) |
| Hippocampus | -0.010 (-0.103, 0.082) | -0.227 (-0.392, -0.062) | 0.206 (0.041, 0.372) | 0.016 (-0.066, 0.098) | -0.158 (-0.305, -0.010) | 0.190 (0.042, 0.337) |
| Fusiform | 0.014 (-0.158, 0.186) | -0.390 (-0.698, -0.082) | 0.418 (0.110, 0.726) | 0.052 (-0.123, 0.228) | -0.320 (-0.636, -0.004) | 0.424 (0.108, 0.741) |
| Inferior Temporal | 0.017 (-0.122, 0.157) | -0.310 (-0.560, -0.061) | 0.345 (0.095, 0.595) | 0.034 (-0.125, 0.194) | -0.304 (-0.592, -0.017) | 0.373 (0.085, 0.661) |
| Rostral Middle Frontal | 0.117 (-0.082, 0.316) | -0.350 (-0.706, 0.006) | 0.584 (0.228, 0.940) | 0.166 (-0.027, 0.359) | -0.243 (-0.590, 0.105) | 0.575 (0.228, 0.923) |
| Lateral Occipital | 0.139 (-0.162, 0.439) | -0.566 (-1.103, -0.029) | 0.843 (0.306, 1.381) | 0.097 (-0.242, 0.435) | -0.622 (-1.232, -0.012) | 0.815 (0.205, 1.425) |
| Precuneus | 0.018 (-0.075, 0.112) | -0.201 (-0.369, -0.034) | 0.238 (0.070, 0.405) | 0.037 (-0.062, 0.136) | -0.173 (-0.352, 0.005) | 0.247 (0.068, 0.425) |
| Insula | 0.034 (-0.038, 0.107) | -0.136 (-0.267, -0.006) | 0.205 (0.075, 0.335) | 0.039 (-0.047, 0.125) | -0.143 (-0.298, 0.012) | 0.221 (0.067, 0.376) |
| Precentral | 0.094 (-0.034, 0.222) | -0.205 (-0.434, 0.023) | 0.394 (0.165, 0.623) | 0.120 (-0.014, 0.254) | -0.164 (-0.405, 0.077) | 0.404 (0.163, 0.646) |
| Meta Temporal | 0.012 (-0.129, 0.154) | -0.320 (-0.573, -0.067) | 0.344 (0.091, 0.597) | 0.029 (-0.133, 0.191) | -0.314 (-0.606, -0.023) | 0.373 (0.081, 0.664) |

| DVR (non-PVC) | | | | | | |
|---|---|---|---|---|---|---|
| **Region** | **Bias (all subjects)** | **Lower LOA (all subjects)** | **Upper LOA (all subjects)** | **Bias (no AD subject)** | **Lower LOA (no AD subject)** | **Upper LOA (no AD subject)** |
| Entorhinal | -0.004 (-0.065, 0.057) | -0.148 (-0.257, -0.038) | 0.139 (0.030, 0.249) | 0.008 (-0.057, 0.073) | -0.130 (-0.247, -0.013) | 0.146 (0.028, 0.263) |
| Amygdala | -0.006 (-0.038, 0.026) | -0.081 (-0.137, -0.024) | 0.068 (0.011, 0.125) | -0.002 (-0.038, 0.034) | -0.078 (-0.143, -0.013) | 0.074 (0.010, 0.139) |
| Hippocampus | -0.010 (-0.058, 0.039) | -0.123 (-0.210, -0.037) | 0.104 (0.017, 0.191) | 0.004 (-0.041, 0.048) | -0.091 (-0.171, -0.011) | 0.098 (0.018, 0.178) |
| Fusiform | -0.025 (-0.118, 0.068) | -0.243 (-0.410, -0.077) | 0.193 (0.027, 0.360) | 0.007 (-0.060, 0.073) | -0.134 (-0.253, -0.014) | 0.147 (0.028, 0.266) |
| Inferior Temporal | -0.028 (-0.092, 0.036) | -0.178 (-0.293, -0.064) | 0.122 (0.008, 0.237) | -0.010 (-0.067, 0.047) | -0.131 (-0.234, -0.028) | 0.111 (0.008, 0.214) |
| Rostral Middle Frontal | 0.031 (-0.046, 0.109) | -0.150 (-0.289, -0.012) | 0.213 (0.074, 0.351) | 0.043 (-0.043, 0.129) | -0.140 (-0.296, 0.015) | 0.226 (0.070, 0.382) |
| Lateral Occipital | 0.060 (-0.079, 0.199) | -0.266 (-0.514, -0.017) | 0.386 (0.138, 0.634) | 0.042 (-0.116, 0.199) | -0.292 (-0.576, -0.009) | 0.376 (0.092, 0.660) |
| Precuneus | -0.019 (-0.086, 0.048) | -0.177 (-0.297, -0.056) | 0.139 (0.018, 0.259) | 0.007 (-0.023, 0.038) | -0.057 (-0.112, -0.002) | 0.072 (0.017, 0.127) |
| Insula | -0.000 (-0.033, 0.032) | -0.076 (-0.133, -0.018) | 0.075 (0.018, 0.132) | 0.008 (-0.023, 0.039) | -0.058 (-0.113, -0.002) | 0.073 (0.018, 0.129) |
| Precentral | 0.017 (-0.031, 0.064) | -0.094 (-0.178, -0.010) | 0.127 (0.043, 0.212) | 0.028 (-0.018, 0.074) | -0.069 (-0.151, 0.014) | 0.125 (0.043, 0.208) |
| Meta Temporal | 0.010 (-0.027, 0.048) | -0.077 (-0.144, -0.010) | 0.098 (0.031, 0.165) | 0.008 (-0.036, 0.052) | -0.086 (-0.165, -0.006) | 0.101 (0.022, 0.180) |
| **DVR (PVC)** | | | | | | |
| **Region** | **Bias (all subjects)** | **Lower LOA (all subjects)** | **Upper LOA (all subjects)** | **Bias (no AD subject)** | **Lower LOA (no AD subject)** | **Upper LOA (no AD subject)** |
| Entorhinal | 0.004 (-0.090, 0.098) | -0.217 (-0.385, -0.048) | 0.225 (0.056, 0.393) | 0.017 (-0.089, 0.123) | -0.208 (-0.399, -0.017) | 0.242 (0.051, 0.433) |
| Amygdala | -0.014 (-0.081, 0.053) | -0.171 (-0.291, -0.051) | 0.144 (0.024, 0.263) | -0.013 (-0.093, 0.067) | -0.183 (-0.327, -0.039) | 0.156 (0.012, 0.301) |
| Hippocampus | 0.001 (-0.101, 0.103) | -0.239 (-0.421, -0.056) | 0.241 (0.058, 0.423) | 0.034 (-0.047, 0.114) | -0.137 (-0.281, 0.008) | 0.204 (0.059, 0.348) |
| Fusiform | -0.055 (-0.207, 0.097) | -0.411 (-0.682, -0.140) | 0.301 (0.029, 0.572) | 0.003 (-0.071, 0.078) | -0.155 (-0.289, -0.020) | 0.161 (0.027, 0.296) |
| Inferior Temporal | -0.041 (-0.135, 0.053) | -0.261 (-0.430, -0.093) | 0.180 (0.012, 0.348) | -0.015 (-0.102, 0.071) | -0.199 (-0.355, -0.043) | 0.168 (0.012, 0.324) |
| Rostral Middle Frontal | 0.096 (-0.029, 0.221) | -0.197 (-0.421, 0.027) | 0.389 (0.166, 0.613) | 0.061 (-0.050, 0.172) | -0.175 (-0.375, 0.025) | 0.297 (0.096, 0.497) |
| Lateral Occipital | 0.149 (-0.194, 0.492) | -0.655 (-1.268, -0.042) | 0.953 (0.340, 1.566) | 0.086 (-0.283, 0.454) | -0.695 (-1.359, -0.032) | 0.866 (0.203, 1.530) |
| Precuneus | -0.031 (-0.198, 0.137) | -0.424 (-0.723, -0.124) | 0.362 (0.062, 0.661) | 0.024 (-0.101, 0.150) | -0.242 (-0.468, -0.016) | 0.291 (0.064, 0.517) |
| Insula | 0.058 (-0.065, 0.181) | -0.231 (-0.451, -0.011) | 0.347 (0.127, 0.567) | 0.074 (-0.066, 0.214) | -0.222 (-0.474, 0.029) | 0.371 (0.119, 0.622) |
| Precentral | 0.077 (0.010, 0.143) | -0.079 (-0.198, 0.040) | 0.233 (0.114, 0.351) | 0.091 (0.024, 0.159) | -0.052 (-0.174, 0.070) | 0.235 (0.113, 0.357) |
| Meta Temporal | -0.028 (-0.136, 0.080) | -0.282 (-0.475, -0.089) | 0.225 (0.032, 0.418) | -0.008 (-0.124, 0.108) | -0.253 (-0.462, -0.045) | 0.237 (0.029, 0.446) |

| R1 (non-PVC) | | | | | | |
|---|---|---|---|---|---|---|
| **Region** | **Bias (all subjects)** | **Lower LOA (all subjects)** | **Upper LOA (all subjects)** | **Bias (no AD subject)** | **Lower LOA (no AD subject)** | **Upper LOA (no AD subject)** |
| Entorhinal | -0.001 (-0.038, 0.036) | -0.088 (-0.154, -0.021) | 0.086 (0.020, 0.153) | 0.001 (-0.044, 0.045) | -0.093 (-0.173, -0.014) | 0.094 (0.015, 0.174) |
| Amygdala | -0.071 (-0.251, 0.109) | -0.492 (-0.813, -0.171) | 0.351 (0.029, 0.672) | -0.079 (-0.293, 0.134) | -0.532 (-0.916, -0.147) | 0.373 (-0.011, 0.757) |
| Hippocampus | 0.046 (-0.072, 0.164) | -0.231 (-0.442, -0.020) | 0.323 (0.112, 0.534) | 0.057 (-0.080, 0.195) | -0.234 (-0.482, 0.014) | 0.349 (0.101, 0.596) |
| Fusiform | 0.029 (-0.042, 0.099) | -0.136 (-0.262, -0.010) | 0.194 (0.068, 0.320) | 0.037 (-0.045, 0.118) | -0.136 (-0.282, 0.011) | 0.209 (0.063, 0.355) |
| Inferior Temporal | 0.002 (-0.056, 0.060) | -0.134 (-0.238, -0.030) | 0.138 (0.034, 0.242) | 0.008 (-0.058, 0.075) | -0.133 (-0.253, -0.013) | 0.150 (0.030, 0.270) |
| Rostral Middle Frontal | -0.067 (-0.130, -0.004) | -0.215 (-0.328, -0.102) | 0.081 (-0.032, 0.194) | -0.061 (-0.134, 0.013) | -0.217 (-0.349, -0.084) | 0.095 (-0.037, 0.228) |
| Lateral Occipital | 0.053 (-0.044, 0.151) | -0.175 (-0.350, -0.001) | 0.282 (0.108, 0.456) | 0.060 (-0.055, 0.175) | -0.184 (-0.391, 0.023) | 0.303 (0.097, 0.510) |
| Precuneus | -0.002 (-0.039, 0.036) | -0.090 (-0.157, -0.023) | 0.086 (0.019, 0.153) | 0.005 (-0.037, 0.046) | -0.083 (-0.157, -0.009) | 0.092 (0.018, 0.166) |
| Insula | -0.070 (-0.180, 0.041) | -0.328 (-0.525, -0.131) | 0.189 (-0.008, 0.386) | -0.073 (-0.205, 0.058) | -0.351 (-0.588, -0.115) | 0.205 (-0.031, 0.441) |
| Precentral | -0.014 (-0.088, 0.060) | -0.187 (-0.320, -0.055) | 0.160 (0.028, 0.292) | -0.005 (-0.090, 0.080) | -0.186 (-0.339, -0.032) | 0.175 (0.022, 0.329) |
| Meta Temporal | -0.001 (-0.038, 0.036) | -0.088 (-0.154, -0.021) | 0.086 (0.020, 0.153) | 0.001 (-0.044, 0.045) | -0.093 (-0.173, -0.014) | 0.094 (0.015, 0.174) |

| R1 (PVC) | | | | | | |
|---|---|---|---|---|---|---|
| **Region** | **Bias (all subjects)** | **Lower LOA (all subjects)** | **Upper LOA (all subjects)** | **Bias (no AD subject)** | **Lower LOA (no AD subject)** | **Upper LOA (no AD subject)** |
| Entorhinal | 0.014 (-0.029, 0.058) | -0.087 (-0.165, -0.010) | 0.116 (0.039, 0.194) | 0.016 (-0.036, 0.068) | -0.093 (-0.186, -0.000) | 0.125 (0.032, 0.218) |
| Amygdala | -0.014 (-0.086, 0.058) | -0.183 (-0.312, -0.054) | 0.155 (0.026, 0.284) | -0.017 (-0.103, 0.068) | -0.199 (-0.353, -0.045) | 0.164 (0.010, 0.318) |
| Hippocampus | 0.099 (-0.035, 0.233) | -0.216 (-0.455, 0.024) | 0.413 (0.173, 0.652) | 0.112 (-0.044, 0.268) | -0.219 (-0.499, 0.062) | 0.442 (0.161, 0.723) |
| Fusiform | 0.025 (-0.023, 0.073) | -0.087 (-0.173, -0.002) | 0.137 (0.052, 0.223) | 0.033 (-0.020, 0.086) | -0.079 (-0.173, 0.016) | 0.145 (0.050, 0.239) |
| Inferior Temporal | -0.053 (-0.112, 0.006) | -0.190 (-0.295, -0.086) | 0.084 (-0.020, 0.189) | -0.052 (-0.122, 0.018) | -0.200 (-0.326, -0.074) | 0.096 (-0.029, 0.222) |
| Rostral Middle Frontal | -0.126 (-0.234, -0.018) | -0.379 (-0.572, -0.186) | 0.127 (-0.066, 0.320) | -0.119 (-0.246, 0.009) | -0.389 (-0.618, -0.160) | 0.151 (-0.078, 0.381) |
| Lateral Occipital | 0.132 (-0.026, 0.290) | -0.238 (-0.521, 0.044) | 0.503 (0.220, 0.785) | 0.147 (-0.038, 0.331) | -0.244 (-0.575, 0.088) | 0.537 (0.206, 0.869) |
| Precuneus | -0.008 (-0.063, 0.047) | -0.137 (-0.235, -0.038) | 0.121 (0.023, 0.219) | 0.001 (-0.060, 0.062) | -0.128 (-0.237, -0.018) | 0.130 (0.020, 0.240) |
| Insula | -0.006 (-0.107, 0.096) | -0.244 (-0.426, -0.062) | 0.233 (0.051, 0.415) | 0.001 (-0.120, 0.121) | -0.255 (-0.471, -0.038) | 0.256 (0.039, 0.472) |
| Precentral | -0.027 (-0.135, 0.080) | -0.279 (-0.471, -0.087) | 0.225 (0.033, 0.417) | -0.016 (-0.140, 0.109) | -0.279 (-0.502, -0.055) | 0.248 (0.024, 0.471) |
| Meta Temporal | 0.016 (-0.105, 0.138) | -0.269 (-0.486, -0.051) | 0.301 (0.084, 0.518) | 0.023 (-0.121, 0.167) | -0.282 (-0.541, -0.023) | 0.328 (0.069, 0.587) |
